# Bridging Developmental Boundaries: Lifelong Dietary Patterns Modulate Life Histories in a Parthenogenetic Insect

**DOI:** 10.1371/journal.pone.0111654

**Published:** 2014-11-03

**Authors:** Alison M. Roark, Karen A. Bjorndal

**Affiliations:** 1 Department of Biology, Furman University, Greenville, South Carolina, United States of America; 2 Department of Biology, University of Florida, Gainesville, Florida, United States of America; University of Cincinnati, United States of America

## Abstract

Determining the effects of lifelong intake patterns on performance is challenging for many species, primarily because of methodological constraints. Here, we used a parthenogenetic insect (*Carausius morosus*) to determine the effects of limited and unlimited food availability across multiple life-history stages. Using a parthenogen allowed us to quantify intake by juvenile and adult females and to evaluate the morphological, physiological, and life-history responses to intake, all without the confounding influences of pair-housing, mating, and male behavior. In our study, growth rate prior to reproductive maturity was positively correlated with both adult and reproductive lifespans but negatively correlated with total lifespan. Food limitation had opposing effects on lifespan depending on when it was imposed, as it protracted development in juveniles but hastened death in adults. Food limitation also constrained reproduction regardless of when food was limited, although decreased fecundity was especially pronounced in individuals that were food-limited as late juveniles and adults. Additional carry-over effects of juvenile food limitation included smaller adult size and decreased body condition at the adult molt, but these effects were largely mitigated in insects that were switched to ad libitum feeding as late juveniles. Our data provide little support for the existence of a trade-off between longevity and fecundity, perhaps because these functions were fueled by different nutrient pools. However, insects that experienced a switch to the limited diet at reproductive maturity seem to have fueled egg production by drawing down body stores, thus providing some evidence for a life-history trade-off. Our results provide important insights into the effects of food limitation and indicate that performance is modulated by intake both within and across life-history stages.

## Introduction

Many morphological, physiological, and life-history traits are shaped by when and to what extent resources are acquired, assimilated, and allocated to various functions. Even in the absence of genetic variation, the expression of these traits can differ among individuals of a species [1]. Such phenotypic plasticity is particularly apparent when resource availability varies spatially or temporally.

Resource scarcity restricts the capacity for growth, maintenance, and reproduction. Even when resources are plentiful, upper limits on the rates of nutrient intake and uptake constrain performance [2]. According to the principle of allocation, these extrinsic and intrinsic constraints should prevent animals from simultaneously maximizing the allocation of nutrients to all traits that influence fitness, such that increased use of resources for one function necessarily decreases allocation to a different function [3], [4], [5]. Resources should therefore be allocated to functions including growth, development, reproduction, and survival according to priority rules [6], [7], [8].

The principle of allocation has generated a number of testable predictions about how animals optimize their use of limited resources according to these priority rules. These predictions almost always include the existence of negative correlations (or trade-offs) among traits. For example, it has been suggested that forgoing reproduction during periods of food scarcity allows animals to divert available resources into maintenance and storage, thereby increasing starvation resistance and the probability of survival until conditions are more conducive to reproduction [9], [10]. As predicted, some food-limited adults do suppress reproduction and survive longer [11], [12].

Trade-offs are also apparent when food limitation occurs early in life. For example, food-limited juveniles often delay developmental transitions to extend the time available for growth [13]. Individuals thus mature at older ages, potentially shortening the reproductive lifespan and lengthening generation time. Alternatively, size thresholds for life-history transitions may shift downward in food-limited juveniles to reduce the demographic costs of extended development (e.g., predation or starvation of juveniles) [14], thus yielding smaller, potentially less fecund, adults [15]. On the other hand, when resources are plentiful, growth and development are accelerated such that both age at maturity and generation time are minimized while adult size, fecundity, and relative fitness are maximized [16], [17].

On the surface, this evidence supports the notions that a) resources allocated to growth or maintenance necessarily diminish the resources available for reproduction, and b) reproduction is inherently costly with respect to survival [18], [19]. Presumably, then, food limitation extends lifespan by inhibiting reproduction. However, preventing oogenesis or vitellogenesis does not necessarily improve lifespan [20], [21], [22], and lifespan can be extended by food restriction even in post-reproductive adults [23]. Furthermore, several studies have reported either no correlation [24] or a positive correlation between longevity and fecundity [25], [26], [27].

Whether traits are negatively or positively correlated may also be determined by the lag between acquisition and allocation. When these processes are asynchronous, the magnitude and direction of trait correlations may differ from when these processes occur concomitantly. For example, adult food limitation may have very different effects on the relationship between fecundity and longevity in “income” breeders that provision offspring with nutrients acquired concurrently than in “capital” breeders that rely on stored nutrients for egg production [6], [28], [29], [30]. Additionally, nutritional stress experienced early in life can constrain subsequent reproductive potential, even if conditions improve for adults [31], [32]. Previously food-limited animals may also be more vulnerable to starvation, illness, or predation because of their small size and limited energy reserves [33], [34], [35], [36]. In these cases, the carry-over effects of previous food limitation can lead to positive correlations between body size, fecundity, and survival.

Clearly, resource acquisition and allocation play a significant role in dictating life histories. Because acquisition and allocation patterns change with age [37], [38], [39], and because life-history traits may be expressed only once (e.g., age at maturity), multiple times (e.g., body size at each molt), or continuously throughout life (e.g., metabolic rate) [40], determining the full range of responses to differences in food availability requires manipulation of food intake throughout the entire lifespan. This approach is particularly challenging for long-lived animals with complex life histories. Even in tractable animal models, studies in which food availability is manipulated are complicated by a lack of consistency in the protocols used for feeding and housing of animals.

In feeding trials, diet is manipulated by altering either the quantity or the quality of food offered. In the former case, which is more common in vertebrate feeding trials, intake can be restricted by pair-feeding, intermittent feeding, or reducing the mass of food offered [41], [42]. In insect feeding trials, these approaches are typically used only for adults [43], [44], [45]. As a result, the effects of quantitative, juvenile food limitation in insects are largely unknown. In the latter case, animals are offered ad libitum quantities of chemically defined diets that vary in nutrient density and/or protein:carbohydrate ratio [24], [46], [47], [48]. In many such studies, particularly in insects, individual intake is not quantified [49] (although see [48]), despite the need for such information when assessing trade-offs [7]. Studies in which individual intake has been quantified have provided exciting information about how insects regulate intake to achieve specific “nutrient targets” that maximize survival or fecundity [49], [50], but such regulation may not be possible on more natural diets. Additionally, evaluating the effects of food limitation on fitness requires that females of sexual species be allowed to mate. However, co-housing individuals complicates the quantification of individual intake and can influence longevity and fecundity due to the effects of crowding [51], [52]. To avoid such problems, the production of eggs by virgin females is often used as a measure of fitness despite the fact that mating enhances egg production [53], [54], [55], [56]. For these reasons, life-history responses to lifelong patterns of intake of natural diets are largely unknown, particularly in invertebrates.

To overcome these obstacles, we used a novel approach by evaluating the effects of quantitative food limitation during different life-history stages in a parthenogenetic animal. The Indian stick insect, *Carausius morosus* (Br.) (Phasmatodea, Lonchodinae) reproduces via apomictic parthenogenesis [57], thus permitting us to manipulate the intake of individually housed females while still allowing them to reproduce. Indian stick insects are hemimetabolous and, unlike many insects, do not undergo an ontogenetic diet shift. For this reason, we were able to use the same food source in either limited (L) or unlimited (U) quantities throughout the entire lifespan and thus to test the effects of constant low and high food availabilities across developmental boundaries. We also switched some insects from L to U or from U to L during the juvenile stage or at reproductive maturity to evaluate the effects of a changing environment. We were then able to determine whether the direction and magnitude of the responses to intake changed with age and ontogeny. The specific questions we addressed were:

1. How does the lifelong pattern of intake affect growth, development, and age and size at life-history transitions? Do these effects differ depending on when food is limited?
2. What morphological and physiological parameters are correlated with longevity and fecundity in reproductively active females with different intake histories? Are these measures of performance negatively or positively correlated with each other?
3. Does juvenile intake have carry-over effects in the adult stage, or is adult performance independent of previous intake history?

Throughout the study, we determined how patterns of both juvenile and adult intake affect physiological and life-history parameters including rates of growth and development, age and size at critical life-history transitions, fecundity, and various measures of lifespan. Our expectations were that a) ad libitum-fed insects should grow and develop faster into larger adults that reproduce more and die sooner than food-limited insects, regardless of when food is limited; b) the influence of adult intake on the expression of adult traits will be constrained by carry-over effects of juvenile intake on age, size, and body condition at the adult molt; and c) we should observe life-history trade-offs between pairs of traits including longevity and fecundity, egg size and number, and current and future reproduction.

## Material and Methods

### Animal care

Insects were housed in a quarantine facility in the Department of Biology, University of Florida. Lights were on a 12 h:12 h light:dark cycle. Room temperature averaged 22.5–24.5°C (Fig. S1), and relative humidity averaged 45–55% (Fig. S2) throughout the trial.

Twenty adult female Indian stick insects (*Carausius morosus*) were obtained from the Exploratorium in San Francisco, California. Eggs oviposited by these females were individually incubated in plastic well plates until hatching. The resulting offspring (*n* = 86) were systematically assigned to treatment groups such that the offspring produced by each adult female were distributed among groups.

Insects were maintained individually in plastic cages (29.5 cm×19 cm×19 cm) that were misted daily with deionized water to provide drinking water. Insects were fed discs cut from leaves of English ivy (*Hedera helix*) daily. Biopsy punches (Miltex Instrument Co., Inc.) were used to create discs of different diameters: 2 mm for first instar insects, 3 mm for second instars, 4 mm for third instars, 5 mm for fourth instars, 6 mm for fifth instars, and 8 mm for sixth instars and adults. Samples of leaf discs of each size were dried daily to constant mass at 60°C and weighed, and the dry mass per disc was calculated (Fig. S3).

Initially, leaf discs were all punched from leaves of ivy grown in culture. English ivy was first obtained from a commercial supplier (Benchmark Foliage, Inc., Plymouth, FL). These plants were maintained under a metal halide grow lamp (Sunmaster Cool Deluxe) on a 12 h:12 h light:dark cycle at approximately 21°C. Ivy was watered weekly with deionized water and fertilized monthly using Peter’s Professional all-purpose plant food (20% total N, 20% available phosphate, 20% soluble potash). Ivy was cultured by taking cuttings twice per month. Cuttings were allowed to root in deionized water for two weeks before being planted in Bayer Advanced Garden multi-purpose potting mix. Beginning in week 17 (prior to the first oviposition of all insects) and continuing until the end of the study, leaves used to cut 8-mm discs were obtained from a private wooded lot near the University of Florida. Runners of *H. helix* were collected weekly from this lot and maintained in Bayer Advanced Garden multi-purpose potting mix under the same conditions used for cultured ivy. Leaf discs smaller than 8 mm in diameter were always cut from cultured ivy leaves.

Insects were offered either more leaf discs than they could consume within 24 hours (ad libitum or unlimited food, U) or a limited number of discs (L) equal to 60% of the average daily mass-specific intake by continuously ad libitum-fed insects in the same life-history stage. Stages included each of six juvenile instars (although one insect progressed through a supernumerary instar), adult prior to first oviposition (pre-ov adult), and adult after first oviposition (post-ov adult). Correcting intake for body size on a percent body mass basis is appropriate because metabolic rate scales proportionally with body mass in *C. morosus* [58]. Because mass-specific intake by continuously ad libitum-fed insects declined after first oviposition, the amount of food offered to food-limited adults after first oviposition was decreased proportionally to match this decline. Food-limited insects in all life-history stages almost always consumed all of the discs they were offered each day, except on days immediately preceding a molt.

**Figure 1:**
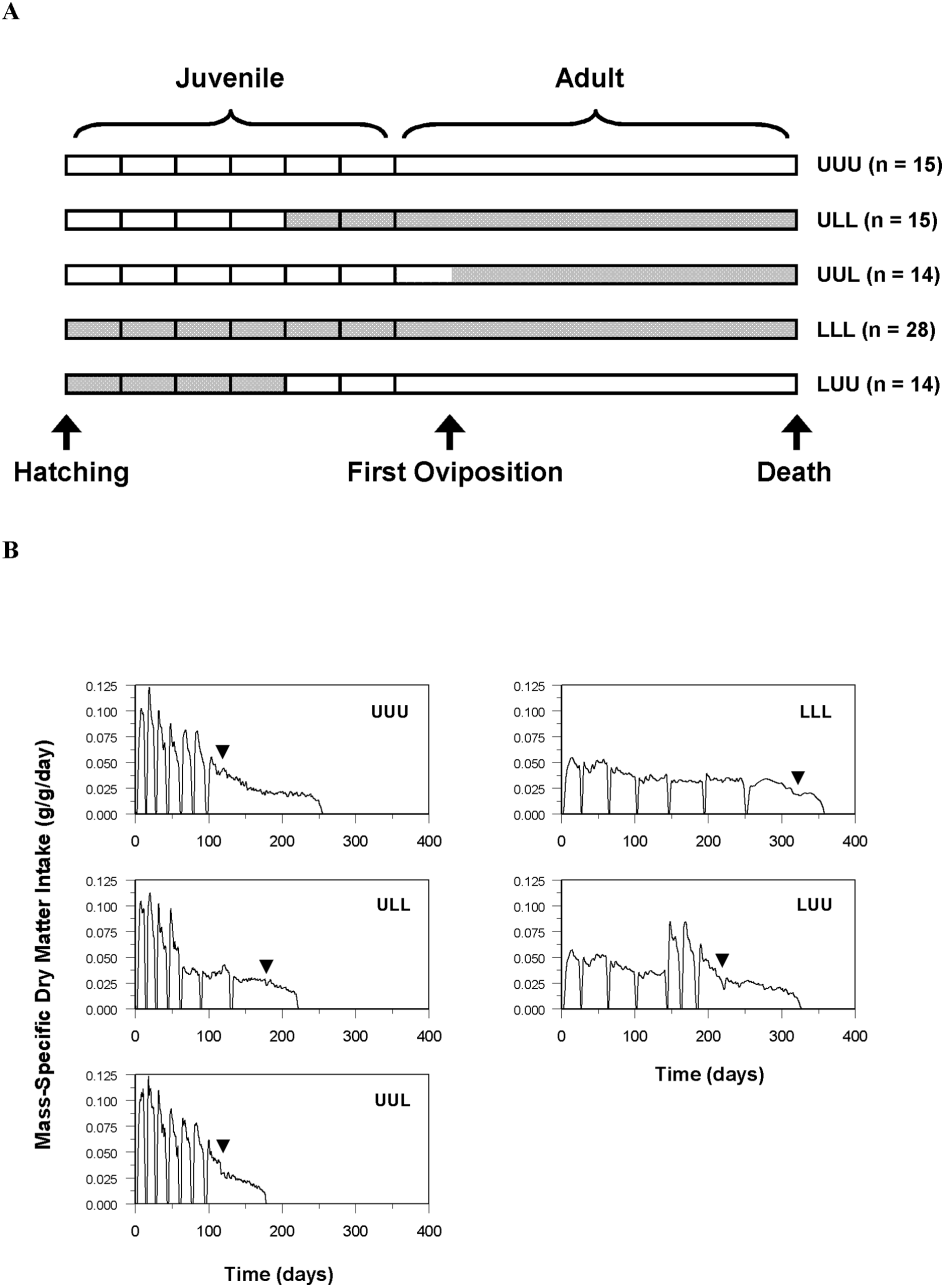
Treatment groups and mass-specific intake. (A) Experimental design. Lifespans are represented by horizontal bars divided into six instars and an adult stage. Time is not to scale, and differences in timing of life-history transitions between groups are not graphically presented. Vertical lines in juvenile stages denote ecdyses. White bars represent life stages when food was offered ad libitum (U, unlimited access to food). Shaded bars represent life stages when food was limited (L) to 60% of the amount of food consumed by insects in group UUU on a percent body mass basis. Because survival to first oviposition was low for insects that were food-limited for the duration of juvenile development, we were unable to test the effects of a diet switch from L to U at first oviposition (LLU). Sample sizes reflect the number of individuals present in each treatment group at the beginning of the trial. (B) Daily mass-specific dry matter intake (g/g/day). Curves were constructed by scaling the duration of each stage for each insect to the average duration of that stage for each treatment group and fitting a loess smoothing function to these data. Points where mass-specific intake declined to zero correspond to ecdyses. The first six time intervals represent juvenile stages; the final time interval represents the adult stage. Arrowheads denote the average age at first oviposition. Mass-specific intake for UUU insects declined after first oviposition. The amount of food offered to food-limited adults after first oviposition was decreased proportionally to match this decline. Sample sizes: UUU *n* = 13, ULL *n* = 13, UUL *n* = 13, LLL *n* = 19 juveniles and 7 adults, LUU *n* = 12.

Leaf discs were offered according to five treatment schedules (Fig. 1A). Acronyms for group names refer to the feeding treatment in three distinct life-history periods: hatch to the end of the fourth instar, the beginning of the fifth instar to first oviposition, and first oviposition to death. For example, the ULL group was fed ad libitum until the end of the fourth instar and was then switched to the limited diet for the remainder of its lifespan. Although the feeding trial initially included a LLU group, we could not test this treatment because survival to reproductive maturity was low (25%) for continuously food-limited juveniles. To ensure a sufficient sample size in the LLL group, all insects (*n* = 7) that were continuously food-limited as juveniles (including those initially in the LLU group) and successfully oviposited as adults were maintained on the limited diet throughout adulthood in group LLL. Unless otherwise noted, the sample sizes indicated in each table and figure include only those individuals that survived through the end of the sixth instar. Given the small number of insects in the LLL group that successfully oviposited, data presented for adult endpoints measured on insects in this group are presented and evaluated with caution.

### Physiological and life-history data

Daily intake by each insect was calculated by determining the number of discs consumed. Partially eaten discs were pressed between microscope slides and scanned, and the surface area of each fragment as a proportion of an uneaten disc was determined using ImageJ (1.37 v). Daily dry matter intake was calculated as the product of discs consumed and dry mass per disc. Daily mass-specific dry matter intake was calculated using estimates of daily body mass computed from periodic body mass measurements, as described below. Frass was collected at the end of each life-history stage.

Samples of each size of leaf disc offered each week were ground with dry ice in a mill (C.W. Brabender Instruments, Inc., South Hackensack, NJ) and dried to constant mass at 60°C. Frass samples were dried to constant mass at 60°C and ground using a mortar and pestle. Nitrogen content of leaf (Fig. S4) and frass samples was determined using a Carlo Erba NA 1500 CNS Elemental Analyzer. Assimilated nitrogen in each life-history stage was calculated as consumed N – frass N, and apparent nitrogen assimilation efficiency (NAE) was then calculated as assimilated N*100/consumed N during each life-history stage.

Insects were weighed weekly and at the end of each life-history stage. The end of an instar was defined as the day on which no leaf discs were consumed prior to a molt. Insects were also photographed at the end of each life-history stage, and body lengths were determined using ImageJ. Measurements of body size at the end of each instar for juvenile UUU insects were then fitted to the allometric equation ln(*y*) = ln(*a*) + *b*ln(*x*), where *y* = body mass and *x* = body length. Relative mass (as an index of body condition) of insects in all treatment groups at the adult molt was calculated as the ratio between actual body mass and body mass predicted by the allometric equation [59]. Specific growth rate (SGR) in each life-history stage was calculated as SGR = 100*(lnBM_f_ – lnBM_i_)/t, where BM_f_ is body mass at the end of a stage, BM_i_ is body mass at the beginning of a stage, and t is the time in that stage.

All eggs oviposited by each insect were weighed. Fecundity was measured as the number of eggs oviposited, and reproductive investment was calculated as the sum wet mass of all eggs oviposited. Reproductive lifespan was calculated as the time between the oviposition of first and last eggs. After death, the number of ovarioles in each insect was determined by dissection. Unfulfilled reproductive potential was measured as the number of eggs in the ovaries after death, and potential fecundity was calculated as unfulfilled reproductive potential + fecundity.

### Statistical analyses

Analysis of variance (ANOVA) was used to test for differences among treatment groups. Data were first tested for normality (Shapiro-Wilk’s test) and homogeneity of variances (Levene’s test) and transformed, if necessary. Pairwise comparisons were evaluated using Tukey’s Honestly Significant Difference post hoc test (if variances were homogeneous) or Tamhane’s T2 post hoc test (if variances were not homogeneous). Data that could not be normalized were analyzed using a Kruskal-Wallis test, and pairwise comparisons were evaluated using Mann-Whitney U tests with α set at 0.005 to account for multiple comparisons. Fecundity was analyzed using ANOVA and also using analysis of covariance with body mass at first oviposition as the covariate. Spearman’s rank correlation test was used to evaluate the strength of the relationship between relative mass at the adult molt and adult lifespan.

Stepwise linear regression was used to determine the factors that best explained variance in fecundity, early egg output (the number of eggs oviposited during the first six days of the reproductive lifespan), and longevity. For these analyses, the dependent variables were fecundity and potential fecundity, number of eggs oviposited in the first six days of the reproductive lifespan, adult lifespan, reproductive lifespan, and total lifespan. Data for seven potential independent variables (body mass, age, stage duration, specific growth rate, mass-specific and total intake, and assimilated nitrogen) in each of the six instars and the pre-oviposition adult stage were tested, along with mass-specific metabolic rates in each life-history stage beginning in the fourth instar (Table S1). Because some insects require a threshold level of food consumption or body stores to initiate reproductive processes [60], [61], the ratio of actual to predicted body mass at the adult molt (as an index of body condition) and both total intake and assimilated nitrogen during a number of multi-stage intervals were also tested as potential independent variables. Variables had to meet a 0.05 significance level to enter a model, and variables with a variance inflation factor (VIF) greater than 10 were excluded from analysis [62]. Normality of the standardized residuals for the most significant of each set of models was confirmed using Shapiro-Wilk’s test.

Least squares linear regression was used to examine the relationships between fecundity and both total and adult lifespan and also to evaluate the strength of the relationship between fecundity and total intake during the reproductive lifespan. Kaplan-Meier survivorship curves were constructed for the entire lifespan (*n* = 86, including all insects used in the study) and for adult lifespan (*n* = 70, including only those individuals that survived through the adult molt). Pairwise comparisons among groups were evaluated using log-rank tests with α set at 0.005 to account for multiple comparisons.

Data were analyzed using SPSS for Windows (Release 11.0.0). S-Plus (Version 7.0) was used for graphing smoothing functions and Kaplan-Meier curves.

## Results

### Intake and nitrogen assimilation efficiency differed among treatments

**Figure 2:**
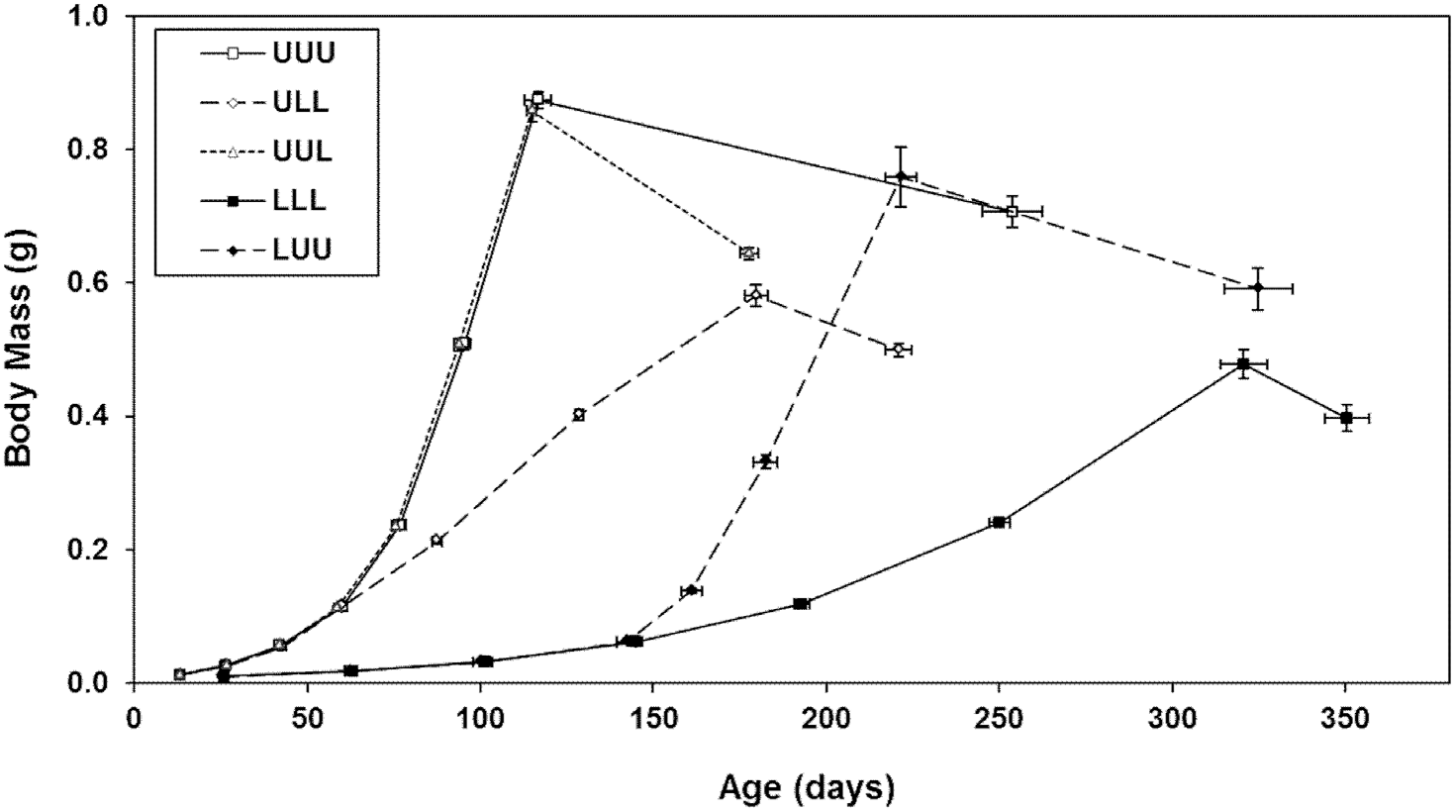
Age and size at each life-history transition. Points represent means (± standard errors) at the end of each instar, at first oviposition, and at death. U = unlimited access to food, L = limited access to food. Sample sizes: UUU *n* = 13, ULL *n* = 13, UUL *n* = 13, LLL *n* = 19 juveniles and 7 adults, LUU *n* = 12. See Table S3 for statistical results for size and age at each point.

Diet treatments yielded different mass-specific intake trajectories among groups. Food-limited insects almost always consumed all of the discs offered each day, which amounted to approximately 60% of the mass-specific intake of ad libitum-fed insects in group UUU in the same life-history stage (Fig. 1B). Total dry matter consumed during each life-history stage except the first instar differed among groups (Table S2). In the second instar, food-limited insects consumed more total dry matter despite being smaller than insects feeding ad libitum because of the longer duration of the instar (Fig. 2 and Table S3). In the third and fourth instars, food-limited insects consumed less total dry matter than insects feeding ad libitum because, although the instar duration was longer, the difference in body mass between food-limited and ad libitum-fed insects was much greater and the limited diet was offered on a mass-specific basis. In subsequent life-history stages, total dry matter consumption was dependent on intake history.

Intake history also affected nitrogen assimilation efficiency (NAE) in all life-history stages. Food-limited insects demonstrated lower NAE than ad libitum-fed insects (Fig. S5), either because of lower nitrogen digestibility or higher nitrogen excretion relative to nitrogen intake than in ad libitum-fed insects.

### Development and growth rates affected age and size at each life-history transition

The duration of each life-history stage differed among groups (Fig. S6). Food-limited insects generally progressed more slowly through each stage than ad libitum-fed insects. Previous intake history affected the duration of the fifth and sixth instars and the pre-oviposition adult stage for ULL and LUU insects, as individuals in these groups progressed through these stages more rapidly than continuously food-limited individuals but more slowly than continuously ad libitum-fed individuals. Insects experiencing food limitation during adulthood prior to first oviposition laid their first eggs later in the adult stage than insects feeding ad libitum during this time. However, duration of adulthood after first oviposition was significantly shorter for insects that experienced food limitation than for insects that were feeding ad libitum during this time, regardless of when the food limitation was initiated. The senescent lifespan, or the duration of the post-reproductive adult stage, did not differ significantly among groups (*F_4,53_* = 0.179, *p* = 0.948). Differences in stage duration are reflected in the percentage of the total lifespan spent as juveniles and adults (Fig. S7).

Food-limited insects also grew more slowly than insects feeding ad libitum (Fig. S8). After a switch from limited to ad libitum feeding at the beginning of the fifth instar, specific growth rates of LUU insects through the final two instars were comparable to those of continuously ad libitum-fed insects. However, during adulthood prior to first oviposition, growth of LUU insects was slower than that of UUU and UUL insects but faster than that of LLL and ULL insects. Insects that experienced a switch from ad libitum to limited feeding grew faster in both the fifth and sixth instars than insects that were continuously food-limited. All insects lost body mass between first oviposition and death. UUL insects lost proportionally more body mass than UUU insects, but all other pairwise comparisons of adult growth rates after first oviposition were not significant.

Body mass did not differ among groups at hatching (*F_4,65_* = 1.00, *p* = 0.414). Mean body mass and mean age at the end of each life-history stage differed among groups (Fig. 2 and Table S3). Body mass was greater and molting occurred at younger ages in ad libitum-fed insects than in food-limited insects in each of the first four instars. At the end of subsequent instars and at first oviposition, body mass and age differed for all groups except UUU and UUL. At death, body mass of UUL insects was not different from body mass of UUU and LUU insects, but all other pairwise comparisons of size were significant. Mean age at death differed among all groups except LLL and LUU.

### Body condition at the adult molt was correlated with adult lifespan

Least squares regression of body mass (*y*) and length (*x*) for UUU insects at the end of each instar yielded the equation ln(*y*) = 2.7112*ln(*x*)–12.018 (*F_1,76_* = 14077.66, *p*<0.0001, *R^2^* = 0.995). This equation was used to calculate predicted body masses for insects in all treatment groups using actual body lengths at the adult molt. The ratio of actual to predicted body mass (as an index of body condition) at the adult molt differed among groups (Table S4). Insects that were food limited in the final two instars (groups LLL and ULL) had significantly smaller relative body masses at the adult molt than those that were ad libitum-fed in the final two instars.

Relative but not absolute body mass at the adult molt was significantly and positively correlated with adult lifespan (*ρ* = 0.269, *p* = 0.041 for relative body mass; *ρ* = −0.036, *p* = 0.790 for absolute body mass). Because the diet was switched at first oviposition for UUL insects, these individuals may have experienced a mismatch among body condition at the adult molt, early egg output, and intake during reproductive activity. To determine whether this mismatch confounded the influence of body condition on adult lifespan, we excluded these insects and re-analyzed the correlations between adult lifespan and both relative and absolute body mass at the adult molt. In these cases, the *p*-values decreased and both relationships were significant (*ρ* = 0.523, *p*<0.001 for the correlation between adult lifespan and relative body mass; *ρ* = 0.323, *p* = 0.030 for the correlation between adult lifespan and absolute body mass).

### Juvenile and adult diets influenced fecundity and longevity

**Figure 3:**
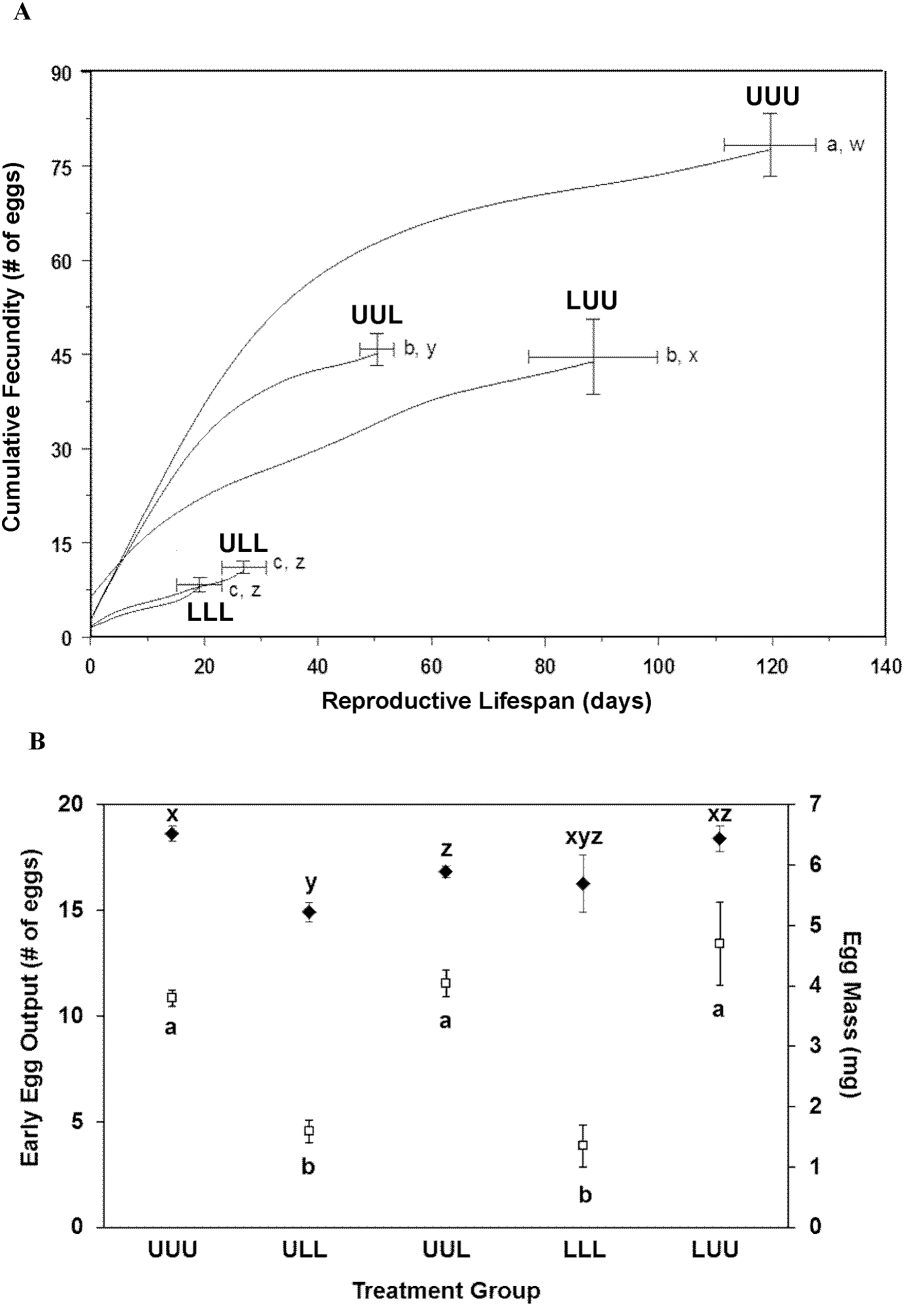
Reproductive performance. (A) Cumulative fecundity of insects in each of five treatment groups. The x-axis represents days of the reproductive lifespan. Each curve terminates at a point corresponding to the mean duration (± standard error) of reproductive activity and the mean fecundity (± standard error) for each group. Curves were constructed by scaling the reproductive lifespan of each insect to the mean reproductive lifespan for that group, determining the mean cumulative fecundity of all insects in that group on each day of the scaled reproductive lifespan, and fitting a smooth spline (*df* = 7) to the resulting means. U = unlimited access to food, L = limited access to food. Sample sizes: UUU *n* = 13, ULL *n* = 13, UUL *n* = 13, LLL *n* = 7, LUU *n* = 12. Different letters to the right of each point indicate significantly different means for fecundity (a, b, and c) and reproductive lifespan (w, x, y, and z) among treatment groups. (B) Metrics of egg production. Early egg output (white squares) was measured as the number of eggs oviposited during the first six days of the reproductive lifespan, and mean egg mass (black diamonds) was measured in mg (means ± standard errors). U = unlimited access to food, L = limited access to food. Sample sizes: UUU *n* = 13, ULL *n* = 13, UUL *n* = 13, LLL *n* = 7, LUU *n* = 12. Different letters indicate significantly different means for early egg output (a, b, and c) and mean egg mass (x, y, and z) among treatment groups.

Fecundity differed among groups (*F_4,53_* = 50.31, *p*<0.0001, Fig. 3A). These differences appeared to result both from differences in reproductive lifespan (*F_4,53_* = 41.70, *p*<0.0001, Fig. 3A) and from differences in early egg output (Fig. 3B). The low fecundity of LLL insects was compounded by low survival to first oviposition. Differences in fecundity did not simply result from differences in body size, as analysis of covariance revealed differences in adjusted mean fecundity when body mass at first oviposition was used as a covariate (Table S4). Insects with different diet histories also laid eggs that differed in size (*F_4,53_* = 8.195, *p*<0.0001, Fig. 3B). Differences in fecundity and egg size did not result from differences in number of ovarioles (Table S4).

Intake history affected the number of eggs remaining in the ovaries at death (*F_4,53_* = 5.286, *p* = 0.001), with LLL insects having a higher unfulfilled reproductive potential than ULL insects (Table S4). All other pairwise comparisons of unfulfilled reproductive potential were not significant. Groups also differed in potential fecundity (*F_4,53_* = 71.62, *p*<0.0001) and total reproductive investment (*F_4,53_* = 49.57, *p*<0.0001). The patterns of potential fecundity and total reproductive investment were comparable to that of fecundity.

**Figure 4:**
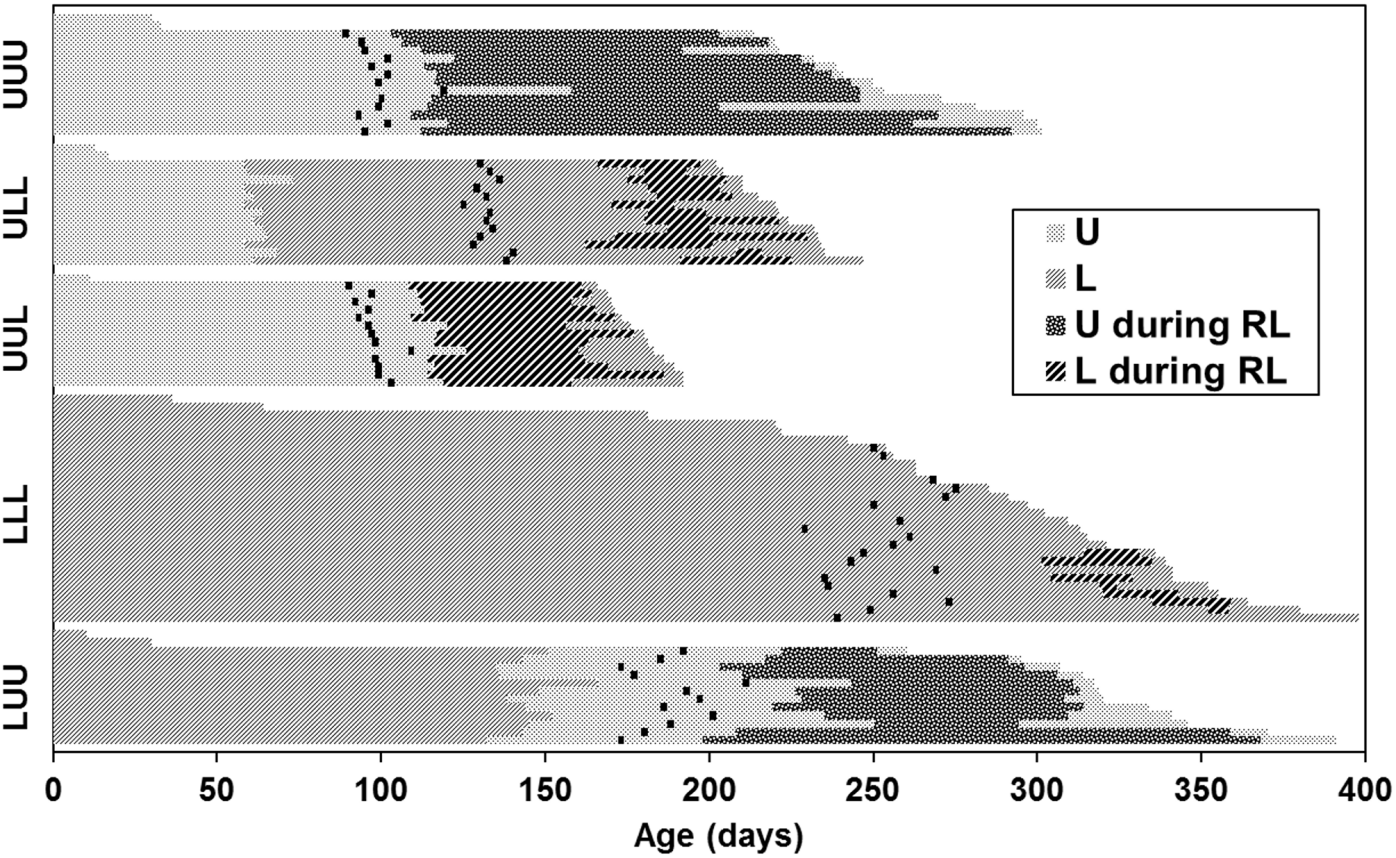
Event history diagram for individual insects maintained on five diet treatments. Each horizontal line represents the lifespan of one individual, with insects in each group arranged in order (top to bottom within a treatment group) from shortest to longest lifespan. U = unlimited access to food, L = limited access to food, RL = reproductive lifespan. The adult molt is indicated by a vertical black line. Data for insects that died during the juvenile stages are included in this diagram but were not included in any analyses except for survivorship curves (Fig. 5). Sample sizes: UUU *n* = 15, ULL *n* = 15, UUL *n* = 14, LLL *n* = 28, LUU *n* = 14.

**Figure 5:**
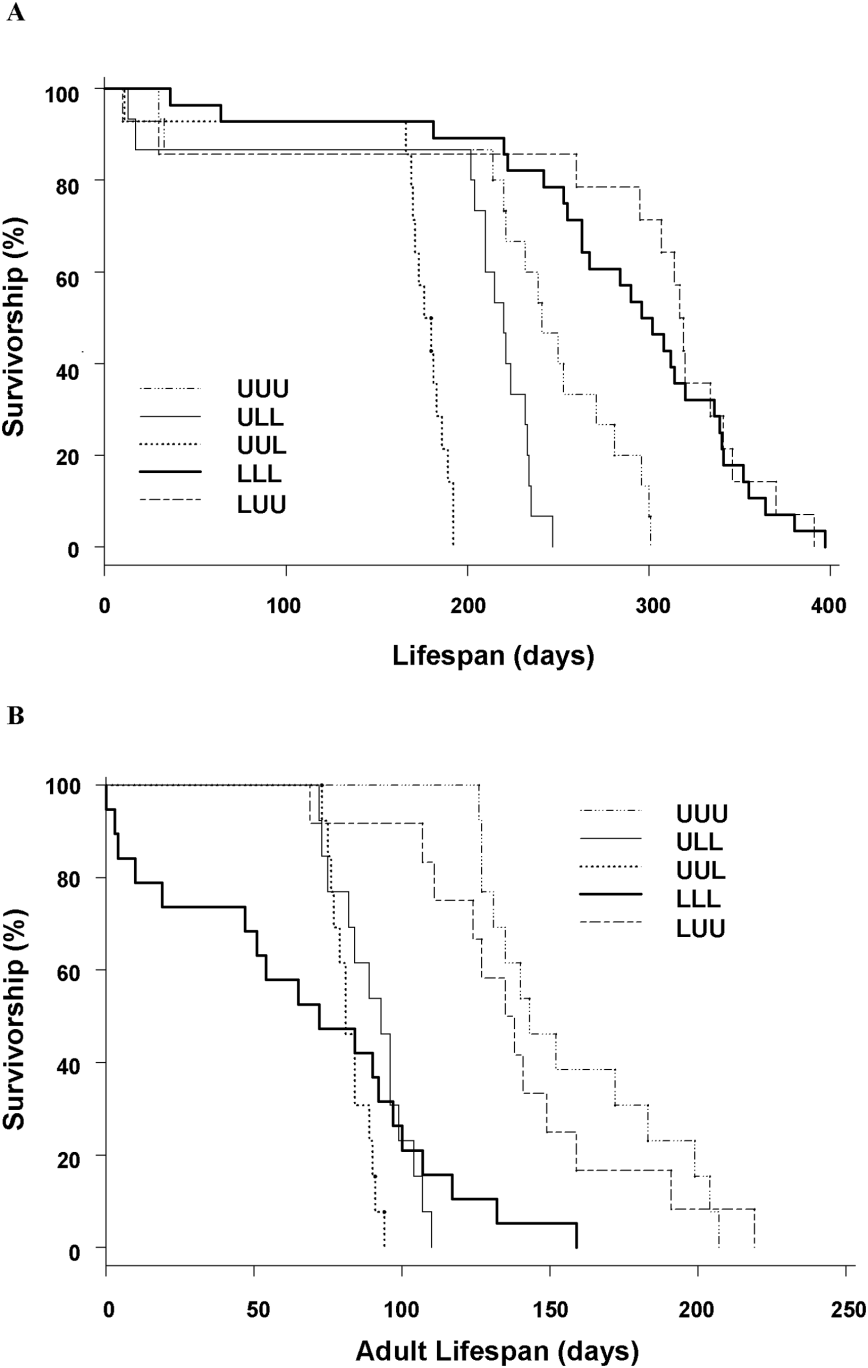
Kaplan-Meier survivorship curves for (A) the entire lifespan and (B) the adult lifespan. Abbreviations: U = unlimited access to food, L = limited access to food. For A, sample sizes are the same as in Figure 4, including insects that died prior to the adult molt (UUU *n* = 15, ULL *n* = 15, UUL *n* = 14, LLL *n* = 28, LUU *n* = 14). For B, only insects that survived to adulthood are included, and sample sizes are UUU *n* = 13, ULL *n* = 13, UUL *n* = 13, LLL *n* = 19 (7 of which oviposited), LUU *n* = 12. For graph A, pairwise log-rank tests indicated that all groups except LLL and LUU differed significantly in longevity. For graph B, UUU and LUU insects had significantly enhanced adult longevity compared to ULL, UUL, and LLL insects.

An event history diagram (sensu [63]) demonstrates the variation in life histories induced by diet treatments (Fig. 4). Pairwise log-rank tests of survival indicated that all groups except LLL and LUU differed in total lifespan (Fig. 5A). This result parallels the ANOVA results for age at death (Table S3). Pairwise log-rank tests of adult lifespan (Fig. 5B) indicated that adult longevity was greater for UUU and LUU insects than for all insects feeding at a limited rate during adulthood.

### Intake and growth determined reproductive performance and longevity

We used stepwise multiple linear regression to identify the most significant predictors of fecundity, early egg output, adult lifespan, reproductive lifespan, and total lifespan (Table 1). Dry matter consumed during the reproductive lifespan was the primary predictor of reproductive output selected by the model, explaining 82.8% of the variance in fecundity (Table 1, model A1, Fig. S9) and 84.7% of the variance in potential fecundity. In addition, growth rate during adulthood prior to first oviposition, average mass-specific intake during the reproductive lifespan, nitrogen assimilated between the beginning of the first instar and first oviposition, and SGR during the fifth instar were also selected as variables in a model that explained a total of 95.3% of the variance in fecundity (Table 1, model A5, *F_5,52_* = 229.84, *p*<0.0001).

**Table 1:** Stepwise multiple linear regression models predicting fecundity (models A1 to A5), initial egg output (models B1 to B4), adult lifespan (models C1 and C2), reproductive lifespan (models D1 and D2), and total lifespan (model E1).

	y	x_1_	x_2_	x_3_	x_4_	x_5_		intercept	B_1_	B_2_	B_3_	B_4_	B_5_	B_6_	*R* ^2^
A1	Fecund	Int RL						9.038	28.05						0.828
A2	Fecund	Int RL	Ad SGR					−4.363	19.09	11.28					0.904
A3	Fecund	Int RL	Ad SGR	MS Int RL				−44.30	17.23	9.583	2051.1				0.929
A4	Fecund	Int RL	Ad SGR	MS Int RL	Nit 1-Ov			−61.89	16.49	6.883	1823.9	1366.5			0.945
A5	Fecund	Int RL	Ad SGR	MS Int RL	Nit 1-Ov	SGR 5		−63.69	17.22	9.875	1651.9	2030.4	−3.837		0.953
B1	6d Eggs	Nit 6-Ov						−9.951	1222.4						0.677
B2	6d Eggs	Nit 6-Ov	SGR 1					−7.575	1264.6	−0.413					0.721
B3	6d Eggs	Nit 6-Ov	SGR 1	Len Ov				−22.44	978.7	−0.926	0.316				0.754
B4	6d Eggs	Nit 6-Ov	SGR 1	Len Ov	MS Int Ad			−21.11	865.6	−0.860	0.258	114.5			0.770
B5	6d Eggs	Nit 6-Ov	SGR 1	Len Ov	MS Int Ad	Nit 5		−24.18	847.1	−0.546	0.371	166.8	−3240.2		0.787
B6	6d Eggs	Nit 6-Ov	SGR 1	Len Ov	MS Int Ad	Nit 5	MR 4	−20.84	777.8	−0.302	0.423	189.0	−3887.9	−0.019	0.811
B7	6d Eggs	Nit 6-Ov	Len Ov	MS Int Ad	Nit 5	MR 4		−17.89	816.8	0.383	212.7	−4840.4	−0.022		0.809
C1	Ad Life	SGR 5						78.00	9.37						0.127
C2	Ad Life	SGR 5	MS Int 3					118.37	11.17	−761.8					0.218
D1	RL	Ad SGR						3.333	29.51						0.455
D2	RL	Ad SGR	SGR 5					−10.93	16.15	10.37					0.483
E1	Tot Life	SGR 3						379.9	−29.89						0.708

Notes: Abbreviations: Fecund = fecundity (number of eggs), 6d Eggs = early egg production (number of eggs oviposited during the first six days of the reproductive lifespan), Ad Life = adult lifespan (days), RL = reproductive lifespan (days), Tot Life = total lifespan (days), Int RL = total dry matter intake (g) during the reproductive lifespan, Nit 6-Ov = nitrogen (g) assimilated between the beginning of the sixth instar and first oviposition, SGR 5 = specific growth rate for body mass (per day) during the fifth instar, Ad SGR = specific growth rate for body mass (per day) during the adult stage prior to first oviposition, SGR 3 = specific growth rate for body mass (per day) during the third instar, SGR 1 = specific growth rate for body mass (per day) during the first instar, Len Ov = body length (mm) at first oviposition, MS Int 3 = average mass-specific intake (g/g) during the third instar, MS Int RL = average mass-specific intake (g/g) during the reproductive lifespan, MS Int Ad = average mass-specific intake (g/g) during adult stage prior to first oviposition, Nit 1-Ov = nitrogen (g) assimilated between the beginning of the first instar and first oviposition, Nit 5 = nitrogen (g) assimilated during the fifth instar, MR 4 = mass-specific metabolic rate (µL/g/hr) in the fourth instar. See Table S1 for the independent variables tested with each dependent variable. Significant independent variables are listed in the order in which they were selected by the models. All models are significant at *p*<0.0001 with *n* = 58 insects.

Early egg output (the number of eggs oviposited during the first six days of the reproductive lifespan) was selected *a posteriori* as a dependent variable because of its apparent dependence on diet and its relationship with both survival and fecundity. Stepwise multiple linear regression identified nitrogen assimilated between the beginning of the sixth instar and first oviposition, body length at first oviposition, average mass-specific intake during adulthood prior to first oviposition, nitrogen assimilated during the fifth instar, and metabolic rate in the fourth instar as significant independent variables in a model that explained a total of 80.9% of the variance in early egg output (Table 1, model B7, *F_5,52_* = 49.37, *p*<0.0001).

Adult lifespan was best predicted by a model (Table 1, model C2, *F_2,55_* = 8.956, *p*<0.001, *R^2^* = 0.218) that included SGR in the fifth instar and mass-specific intake in the third instar as significant independent variables. Reproductive lifespan was best predicted by a model (Table 1, model D2, *F_2,55_* = 27.63, *p*<0.0001, *R^2^* = 0.483) that included SGR during adulthood prior to first oviposition and SGR in the fifth instar as significant independent variables. Total lifespan was best predicted by a model (Table 1, model E1, *F_1,56_* = 139.03, *p*<0.0001, *R^2^* = 0.708) that included a single, negatively correlated variable (SGR in the third instar). The SGRs and mass-specific intakes we tested as potential variables for these models were calculated within but not across life-history stages (Table S1), such that multi-stage patterns of growth and mass-specific intake were not represented as potential independent variables. For this reason, the influence of growth and intake during specific instars on subsequent lifespan should not be overstated. Rather, performance during the third and fifth instars likely represents overall patterns of growth and/or intake prior to or after the juvenile diet switch, respectively.

### Life histories provide little evidence of trade-offs

We found little evidence for a trade-off between reproduction and survival. Total lifespan was not significantly associated with fecundity or potential fecundity (Fig. S10) when data for all groups were combined (*p*>0.5) or when each group was analyzed individually (*p*>0.3 in all cases). On the other hand, adult lifespan was significantly and positively associated with fecundity when data for all treatments were combined (fecundity: *F_1,56_* = 25.67, *p*<0.0001, *R^2^* = 0.302, Fig. S11; potential fecundity: *R^2^* = 0.343). However, individual regressions of fecundity versus adult lifespan for each group were all insignificant (*p*>0.2 in all cases), indicating that no relationship existed between these variables within individual groups.

We also found no evidence for a trade-off between fecundity and egg size. There was neither a significant interaction between average egg mass and fecundity (*p*>0.2) nor an effect of fecundity on average egg mass (*p*>0.3).

## Discussion

Despite the importance of nutrition in determining life histories, few studies have controlled food availability across developmental boundaries to evaluate the effects of lifelong intake patterns. Here, we used a hemimetabolous parthenogen to determine the life-history responses to limited and unlimited availability of a natural diet. Using a hemimetabolous insect that does not undergo an ontogenetic diet shift permitted the use of a homogeneous diet throughout the study, and using a parthenogen allowed us to house our insects individually while monitoring reproductive output.

Our results indicate that life histories are plastic in response to both juvenile and adult food limitation, although the magnitude and direction of the responses differed ontogenetically. Food limitation during developmental and reproductive life-history stages had opposite effects on the rate of progression through each stage, with stage duration increasing in response to food limitation during juvenile and pre-oviposition adult stages and stage duration decreasing in response to food limitation after reproductive maturity. Although developmental delays provided additional time for growth, they were not sufficient to allow full compensation of body size by food-limited juveniles. As a result, insects that experienced juvenile food limitation were both smaller and older at each molt and during adulthood than continuously well fed insects. Adjusting size thresholds downward and age thresholds upward represents a compromise between the need to maximize body size (because of its potential effects on fitness) and the need to minimize the demographic costs of extended development [14].

Although growth slowed in response to food limitation, juvenile insects that experienced a switch from ad libitum to limited feeding grew faster in both the fifth and sixth instars than insects that were continuously food-limited. These insects (in group ULL) also had metabolic rates that were marginally lower than those of LLL insects in the fifth instar [58]. Thus they may have been slightly more efficient in converting their limited incoming nutrients to growth, allowing them to compensate partially for the effects of food limitation.

Food limitation also had drastic negative effects on reproduction, particularly when food was limited during the late juvenile and early adult stages. Plasticity of size thresholds alone does not explain these results, as they persisted even when fecundity was corrected for body mass. Furthermore, differences in fecundity were substantial even among insects of similar size that ate at different rates as adults. Ovary morphology also does not explain these results, as groups did not differ in ovariole number. Rather, reproductive output was likely modulated by a combination of factors including both cumulative and mass-specific adult intake, growth rates during late juvenile and early adult stages (which indirectly influence adult intake through effects on adult body size), and nitrogen assimilated prior to reproductive activity. Of these factors, the total dry mass of food consumed during the reproductive lifespan most significantly influenced reproductive output, with 83% of the variation in fecundity explained by this factor alone.

### Intake predicts fecundity; growth predicts survival

This strong, positive correlation between fecundity and total dry matter intake during the reproductive lifespan indicates an “income” breeding strategy [6], [28], [29], [30], in which the resources allocated to reproduction are acquired primarily during the reproductive period. An income breeding strategy is appropriate for a species like *C. morosus*, in which oogenesis and vitellogenesis are non-cyclic and continuous and in which the ovaries contain primarily immature oocytes immediately after the adult molt [64], [65]. Furthermore, income breeders commonly demonstrate both a positive correlation between longevity and fecundity [66], [67] and a pattern of decreased consumption and production with age [68], both of which were seen in this study. Although income breeders could conceivably mitigate the effects of food limitation on reproductive rate by extending the duration of reproductive activity, food-limited adults in our study had shortened, rather than protracted, reproductive lifespans. Thus, the severely diminished fecundities of LLL and ULL insects likely resulted from both limited access to food and hastened senescence, the combination of which constrained both the resources and time available to generate eggs.

While fecundity was positively correlated with intake during the reproductive lifespan, adult survival was positively correlated with growth rates during late juvenile and immature adult stages and with body composition at the adult molt. Thus, insects that ate and grew the fastest before reproducing were larger, had proportionally greater body stores as adults, and also survived longer as adults than food-limited insects. We therefore demonstrated that adult food limitation had a direct and negative impact on fecundity, whereas juvenile food limitation had a direct and negative impact on adult survival. These data, like those of [43] and [69], suggest that adult-derived “income” fuels egg production while “capital” acquired and stored prior to the onset of reproductive activity fuels subsequent survival, at least in some phytophagous insects.

### Effects of intake on early egg output

Body stores present in early adulthood may also function as an index of food availability that entrains the rate and pattern of egg production at the onset of reproductive activity [60], [70], [71], [72]. Modulating reproductive output in response to internal state is a particularly appropriate strategy for a phytophagous insect whose performance is likely nitrogen-limited [73]. In our study, early egg output was predicted primarily by the quantity of nitrogen assimilated during the late juvenile and early adult stages, although factors such as body size at the onset of reproduction, mass-specific intake during the pre-reproductive adult stage, and juvenile nitrogen intake and metabolic rate were also influential. Given their greater consumption rates, insects feeding ad libitum as late juveniles and pre-reproductive adults had higher body condition scores and likely accumulated proportionally more reserves (including nitrogen) prior to first oviposition, thus permitting a higher early egg output than those feeding at a limited rate.

Prospectively matching reproductive activity to the environment is a logical strategy for an income breeder, but only if the assumption that past and future conditions are correlated holds true. If, however, environmental conditions differ for juveniles and adults, then intake and reproductive rates may be mismatched. In our study, for example, the incongruity between high early egg output and low food availability for UUL insects may have forced these individuals to supplement incoming resources with body stores that could otherwise have been allocated to survival. The exhaustion of these stores would explain the shortened adult lifespan of UUL insects relative to continuously well fed individuals. Two specific results lend support to this suggestion: all UUL individuals were more fecund than expected given their total intake during the reproductive lifespan, and the relationship between relative body mass at the adult molt and adult lifespan for all insects was stronger when UUL insects (the only insects to experience a diet switch as adults) were excluded from the correlation analysis. Thus, we suggest that a mismatch between juvenile and adult intake led to a partial decoupling of the relationship between relative body mass and longevity for insects that experienced a diet switch as adults. In this specific case, a tradeoff between longevity and fecundity may have existed, as resources were preferentially allocated away from survival and toward reproductive output when resource availability changed suddenly at reproductive maturity.

### Food limitation and life histories

Food availability clearly modulates life histories through its effects on growth, development, storage, and reproduction. Whether these effects carry over from one life-history stage to another determines how well individuals compensate for a changing environment. In our study, insects that experienced a switch from low to high food availability during development compensated somewhat for their poor start in life by growing faster, molting at larger sizes, accumulating more body stores, and producing more eggs than insects that were continuously food-limited. However, compensation was incomplete and was not accompanied by catch-up growth or an extended reproductive lifespan relative to continuously ad libitum-fed insects. Thus, life histories were moderately flexible in response to environmental variation, allowing insects to mitigate (albeit only partially) a poor early start once conditions improved. On the other hand, insects that experienced a switch from high to low food availability during development were less able to mitigate the effects of poor conditions. Their slow growth, short reproductive lifespans, limited body stores, and diminished reproductive output are evidence that poor conditions experienced late in development compromise performance more profoundly than similar conditions experienced earlier in life.

The fact that food limitation impaired both reproduction and adult survival indicates that insects feeding ad libitum as adults did not incur mortality costs simply because they reproduced more than food-limited insects. This result contradicts the assumption that food limitation elicits a trade-off between reproduction and survival [18], [19]. Furthermore, we detected no evidence of a trade-off between current and future reproduction or between early fecundity and adult lifespan [74], [75]. Insects feeding ad libitum as pre-reproductive adults exhibited both higher early egg outputs and higher fecundities than those insects that were food-limited during this period.

Reproductive costs (such as increased mortality risk) may differ among species depending on the relative timing of resource acquisition and allocation to reproduction [6]. The existence of a longevity-fecundity trade-off requires that the resources allocated to reproduction and maintenance are derived from a common resource pool and that the utilization of resources from this pool for egg production necessarily decreases the availability of resources for survival [7], [76]. Because insects in our study seem to have allocated body stores primarily to maintenance and diverted incoming resources to egg production, these processes likely did not compete for resources and therefore were not negatively correlated [30].

Our study demonstrates that the life-history responses to intake depend to a large extent on the timing of nutritional stress. Food limitation experienced at any point during life led to decreased fecundity, such that reproductive output was maximized when intake throughout life was also maximized. Total lifespan was maximized when intake and correspondingly growth were limited early in life, but only because food limitation extended development. On the surface, these results seem to provide support for a longevity-fecundity trade-off. However, although insects that were continuously food-restricted experienced both increased longevity and decreased reproductive output relative to continuously ad libitum-fed insects, those individuals that were switched to the limited diet either as juveniles or at reproductive maturity demonstrated deficits in both longevity and fecundity. We also found that intake and growth during juvenile and immature adult stages were positively associated with adult and reproductive lifespans but negatively associated with total lifespan. These data support the contention that a “grow fast and die young” strategy [77] can help to maximize reproductive output when food is readily available. When food is limited, however, ontogeny-dependent costs influence fitness-related traits such as body size, reproductive lifespan, and fecundity. Performance is therefore modulated by nutritional conditions experienced both within and across life-history stages.

## Supporting Information

Figure S1 **Air temperature in the quarantine facility.**
(DOC)

Figure S2 **Relative humidity in the quarantine facility.**
(DOC)

Figure S3 **Leaf disc dry mass.**
(DOC)

Figure S4 **Leaf disc nitrogen content.**
(DOCX)

Figure S5 **Apparent nitrogen assimilation efficiency.**
(DOC)

Figure S6 **Duration of each life-history stage.**
(DOC)

Figure S7 **Percent of the total lifespan comprised of juvenile and adult stages.**
(DOC)

Figure S8 **Specific growth rates.**
(DOC)

Figure S9 **Regression of fecundity and intake during the reproductive lifespan.**
(DOCX)

Figure S10 **Relationship between fecundity and total lifespan.**
(DOCX)

Figure S11 **Relationship between fecundity and adult lifespan.**
(DOCX)

Table S1 **The five dependent and 91 independent variables tested in stepwise linear regression analyses.**
(DOC)

Table S2 **Total leaf dry mass consumed during each life-history stage.**
(DOC)

Table S3 **Statistical results for body mass and age at each life-history transition.**
(DOC)

Table S4 **Body condition at the adult molt, mass-corrected fecundity, number of ovarioles, and unfulfilled reproductive potential.**
(DOC)
