# Supplementary material for: Bridging Developmental Boundaries: Lifelong Dietary Patterns Modulate Life Histories in a Parthenogenetic Insect
Source: PLoS One. 2014 Nov 3;9(11):e111654. doi: 10.1371/journal.pone.0111654 (PMC4218793; doi:10.1371/journal.pone.0111654)
Supplement: Figure S1 — Air temperature in the quarantine facility. (DOC) [file pone.0111654.s001.doc]

**
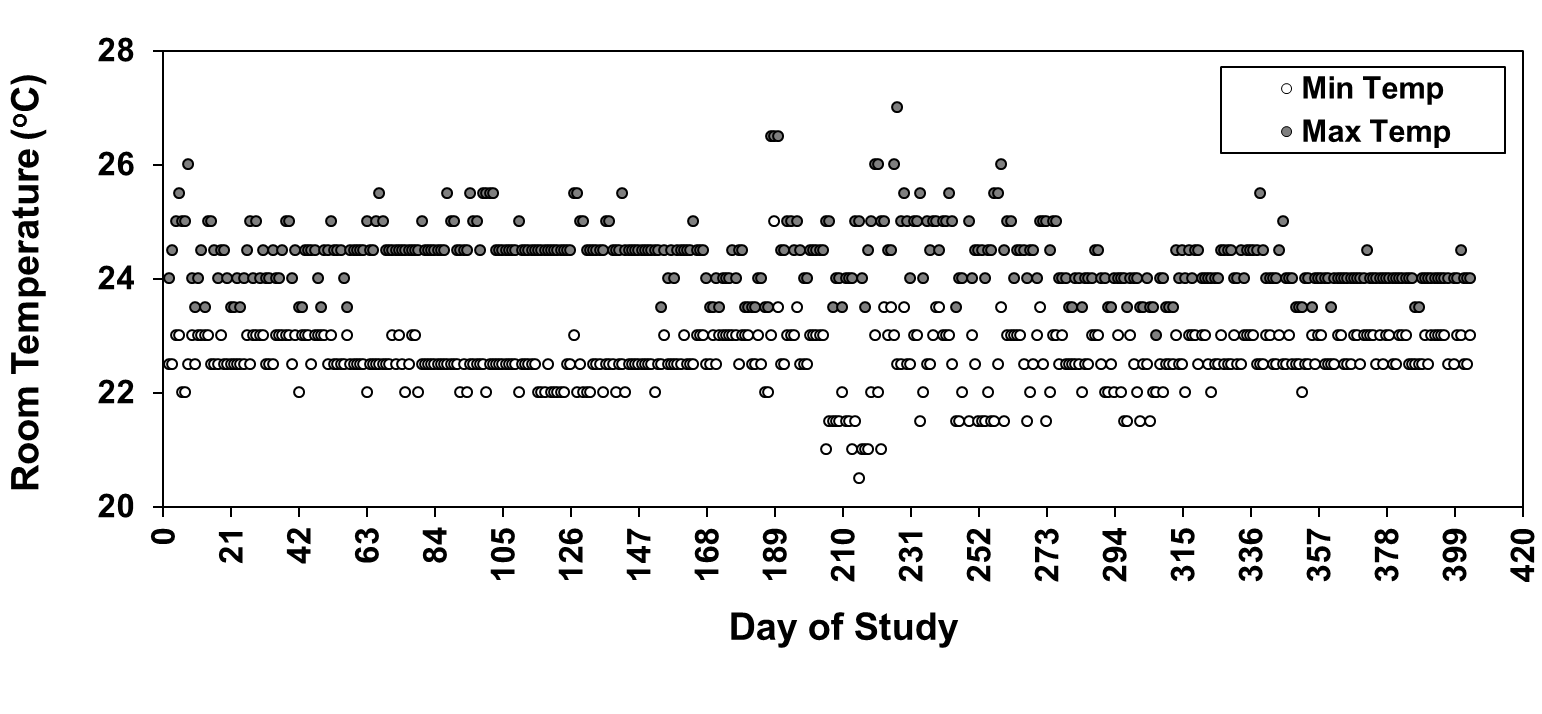
**

Figure S1. Air temperature minima and maxima in the quarantine facility. Temperature and humidity were monitored by two digital min/max thermometers in different locations within the room. Each point represents the mean of two measurements.
