# Supplementary material for: Bridging Developmental Boundaries: Lifelong Dietary Patterns Modulate Life Histories in a Parthenogenetic Insect
Source: PLoS One. 2014 Nov 3;9(11):e111654. doi: 10.1371/journal.pone.0111654 (PMC4218793; doi:10.1371/journal.pone.0111654)
Supplement: Figure S3 — Leaf disc dry mass. (DOC) [file pone.0111654.s003.doc]

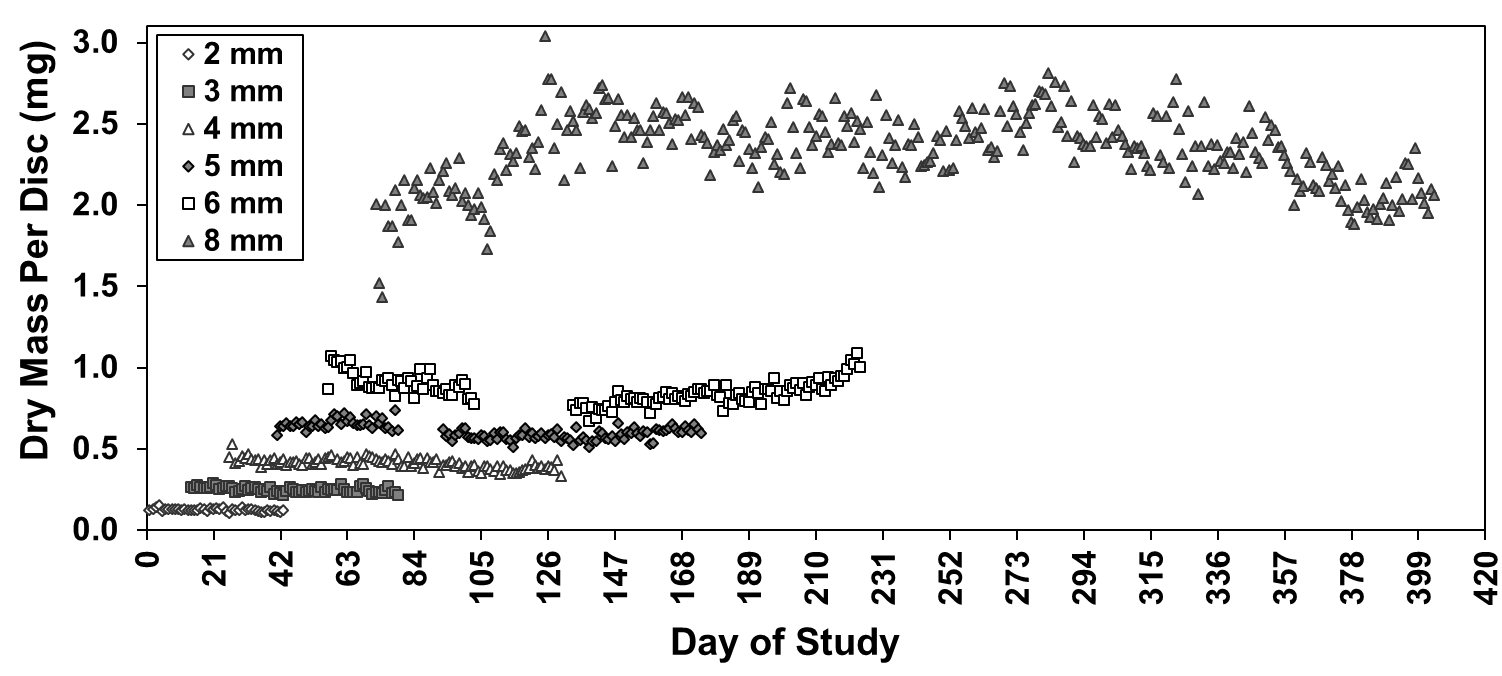


Figure S3. Mean dry mass (mg) of each size of leaf disc offered on each day of the study. Gaps in the data for 5-mm and 6-mm discs represent times when none of the experimental insects was in the fourth and fifth instars, respectively.
