# Supplementary material for: Bridging Developmental Boundaries: Lifelong Dietary Patterns Modulate Life Histories in a Parthenogenetic Insect
Source: PLoS One. 2014 Nov 3;9(11):e111654. doi: 10.1371/journal.pone.0111654 (PMC4218793; doi:10.1371/journal.pone.0111654)
Supplement: Figure S4 — Leaf disc nitrogen content. (DOCX) [file pone.0111654.s004.docx]

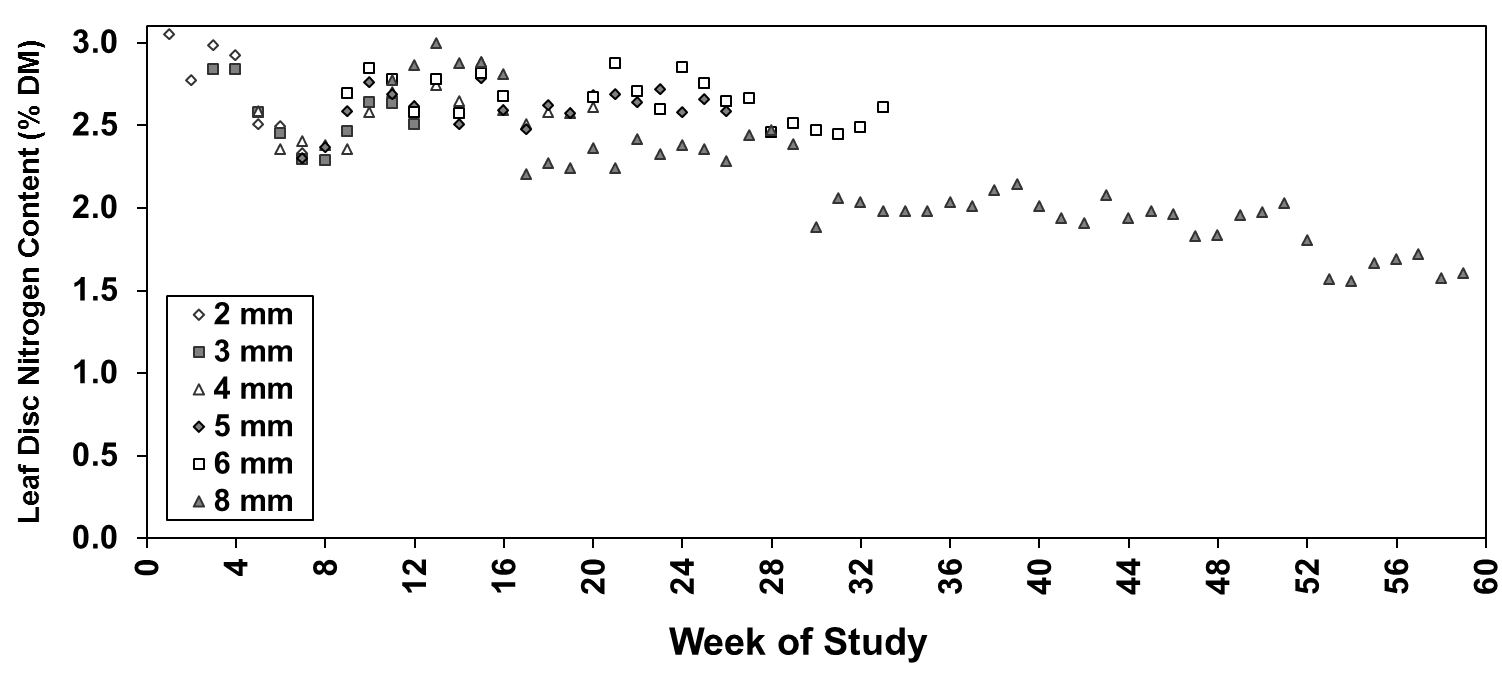


Figure S4. Nitrogen content (% dry matter, DM) of each size of leaf disc offered in each week of the study. Leaf discs collected on each day of the study were pooled for each week, dried to constant mass at 60 °C, and ground in a mill. Nitrogen content was then determined using a Carlo Erba NA 1500 CNS Elemental Analyzer.
