# Supplementary material for: Bridging Developmental Boundaries: Lifelong Dietary Patterns Modulate Life Histories in a Parthenogenetic Insect
Source: PLoS One. 2014 Nov 3;9(11):e111654. doi: 10.1371/journal.pone.0111654 (PMC4218793; doi:10.1371/journal.pone.0111654)
Supplement: Figure S6 — Duration of each life-history stage. (DOC) [file pone.0111654.s006.doc]

**
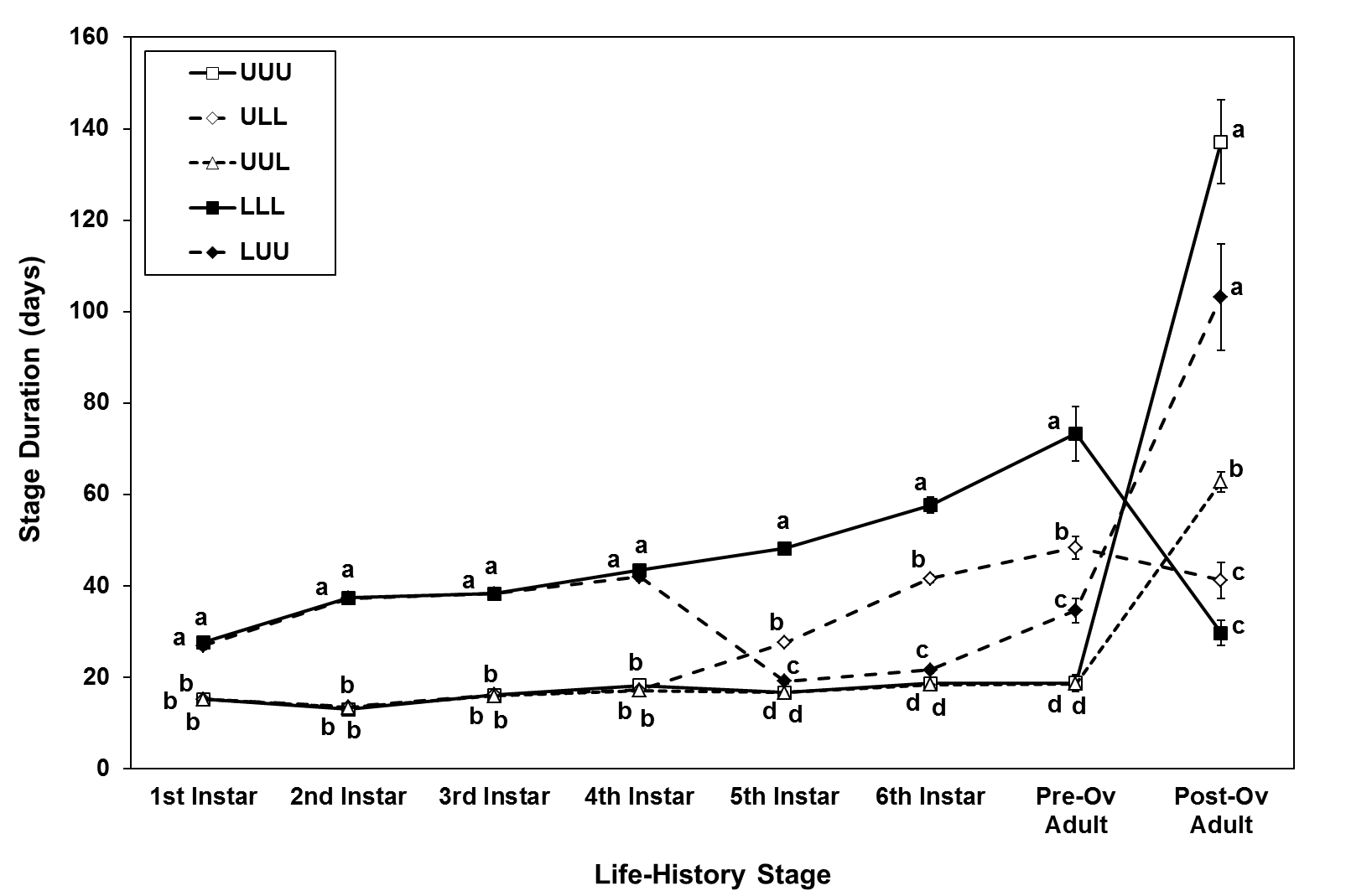
**

Figure S6. Duration of each life-history stage (means ± standard errors). U = unlimited access to food, L = limited access to food. Sample sizes: UUU *n* = 13, ULL *n* = 13, UUL *n* =13, LLL *n* = 19 juveniles and 7 adults, LUU *n* = 12. Means with different letters are significantly different among treatment groups within life-history stages.
