# Supplementary material for: Bridging Developmental Boundaries: Lifelong Dietary Patterns Modulate Life Histories in a Parthenogenetic Insect
Source: PLoS One. 2014 Nov 3;9(11):e111654. doi: 10.1371/journal.pone.0111654 (PMC4218793; doi:10.1371/journal.pone.0111654)
Supplement: Figure S7 — Percent of the total lifespan comprised of juvenile and adult stages. (DOC) [file pone.0111654.s007.doc]

**
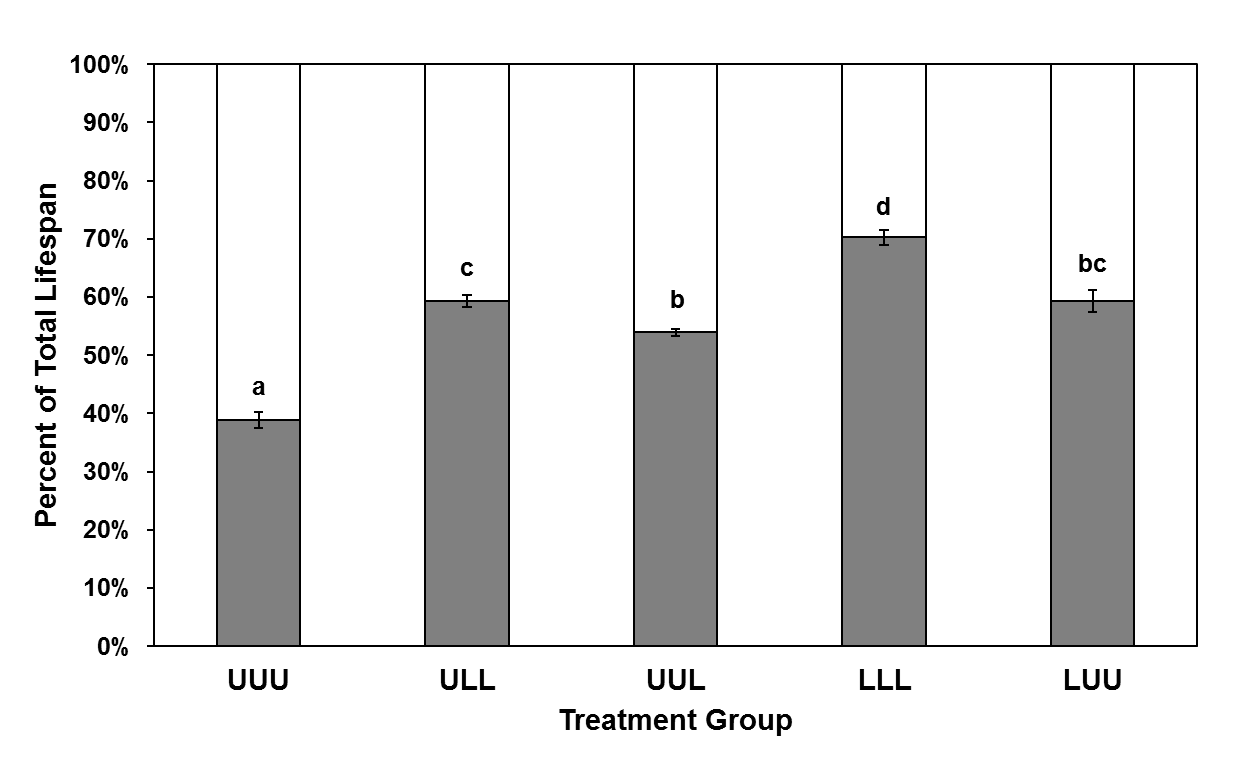
**

Figure S7. Percent of the total lifespan comprised of juvenile (shaded bars) and adult (white bars) stages (means ± standard errors). U = unlimited access to food, L = limited access to food. Sample sizes: UUU *n* = 13, ULL *n* = 13, UUL *n* =13, LLL *n* = 7, LUU *n* = 12. Means with different letters are significantly different among treatment groups.
