# Supplementary material for: Bridging Developmental Boundaries: Lifelong Dietary Patterns Modulate Life Histories in a Parthenogenetic Insect
Source: PLoS One. 2014 Nov 3;9(11):e111654. doi: 10.1371/journal.pone.0111654 (PMC4218793; doi:10.1371/journal.pone.0111654)
Supplement: Figure S9 — Regression of fecundity and intake during the reproductive lifespan. (DOCX) [file pone.0111654.s009.docx]

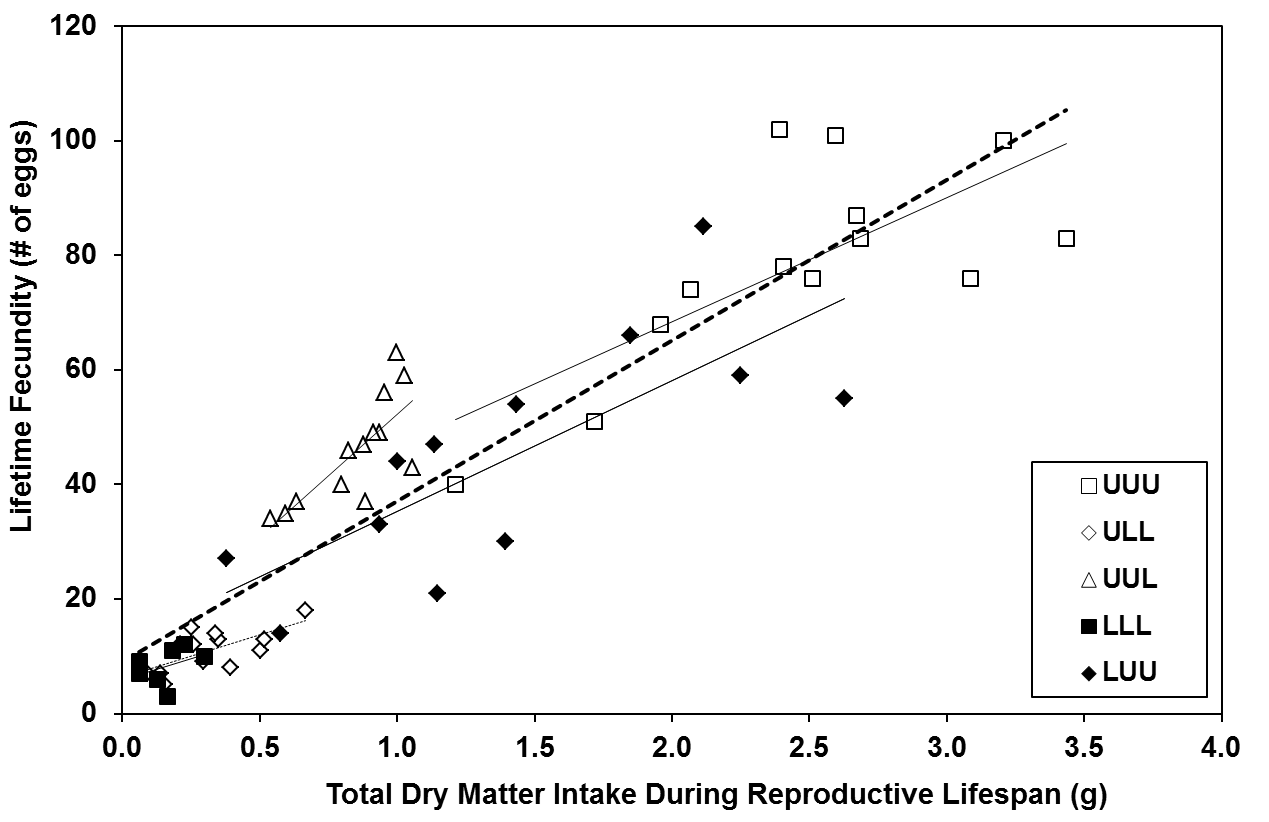


**y = 28.053x + 9.038**

***F_1,56_* = 276.00, *p* < 0.0001,**

**R^2^ = 0.828**

Figure S9. Relationship between fecundity and total dry matter (g) consumed during the reproductive lifespan for all insects that oviposited (*n* = 58) as determined by least squares linear regression. Trendlines depict the relationships within individual groups (thin lines) and among all insects that oviposited (thick hashed line). The equation, F-value, p-value, and R^2^ value included on the figure are the results of the regression analysis of data for all groups combined. U = unlimited access to food, L = limited access to food.
