# Supplementary material for: Bridging Developmental Boundaries: Lifelong Dietary Patterns Modulate Life Histories in a Parthenogenetic Insect
Source: PLoS One. 2014 Nov 3;9(11):e111654. doi: 10.1371/journal.pone.0111654 (PMC4218793; doi:10.1371/journal.pone.0111654)
Supplement: Figure S10 — Relationship between fecundity and total lifespan. (DOCX) [file pone.0111654.s010.docx]

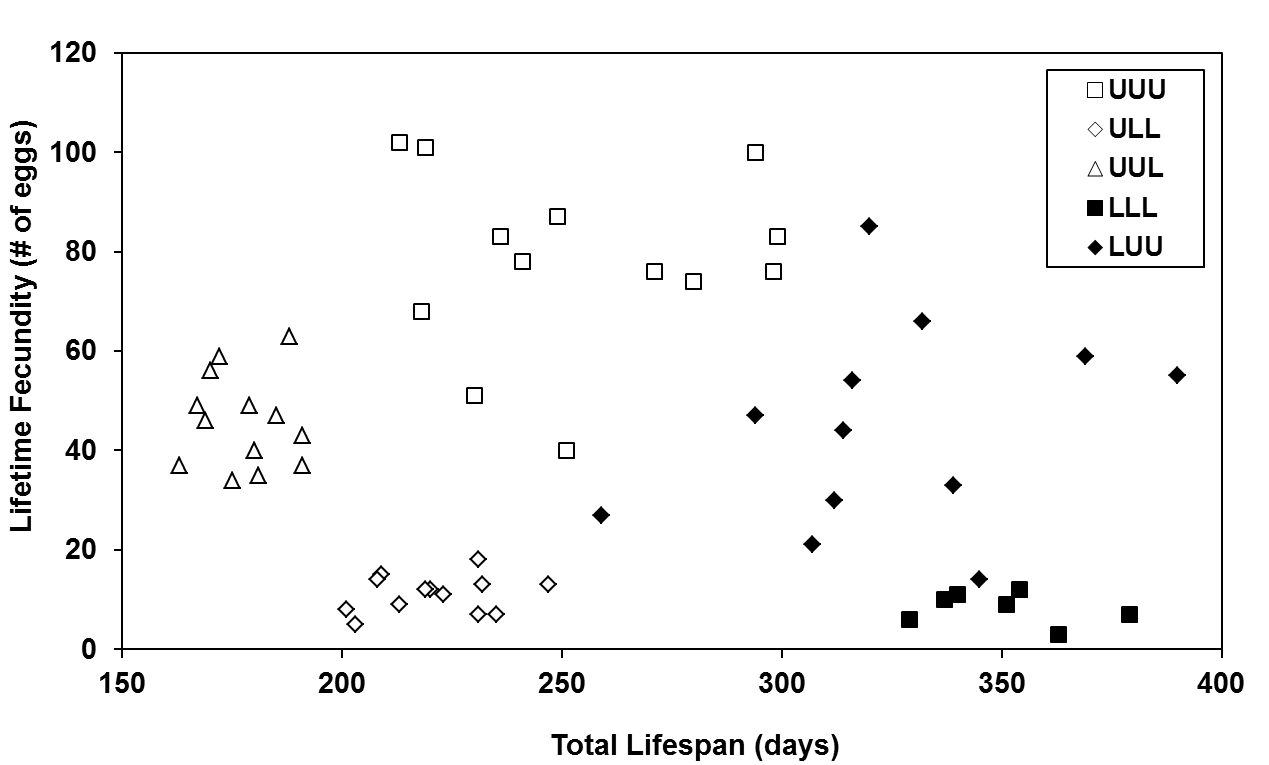


Figure S10. Relationship between fecundity and total lifespan (days) for all insects that oviposited (*n* = 58). Least squares linear regression found no significant association between fecundity and total lifespan when data for all groups were combined or when each group was analyzed individually. U = unlimited access to food, L = limited access to food.
