# Supplementary material for: Bridging Developmental Boundaries: Lifelong Dietary Patterns Modulate Life Histories in a Parthenogenetic Insect
Source: PLoS One. 2014 Nov 3;9(11):e111654. doi: 10.1371/journal.pone.0111654 (PMC4218793; doi:10.1371/journal.pone.0111654)
Supplement: Figure S11 — Relationship between fecundity and adult lifespan. (DOCX) [file pone.0111654.s011.docx]

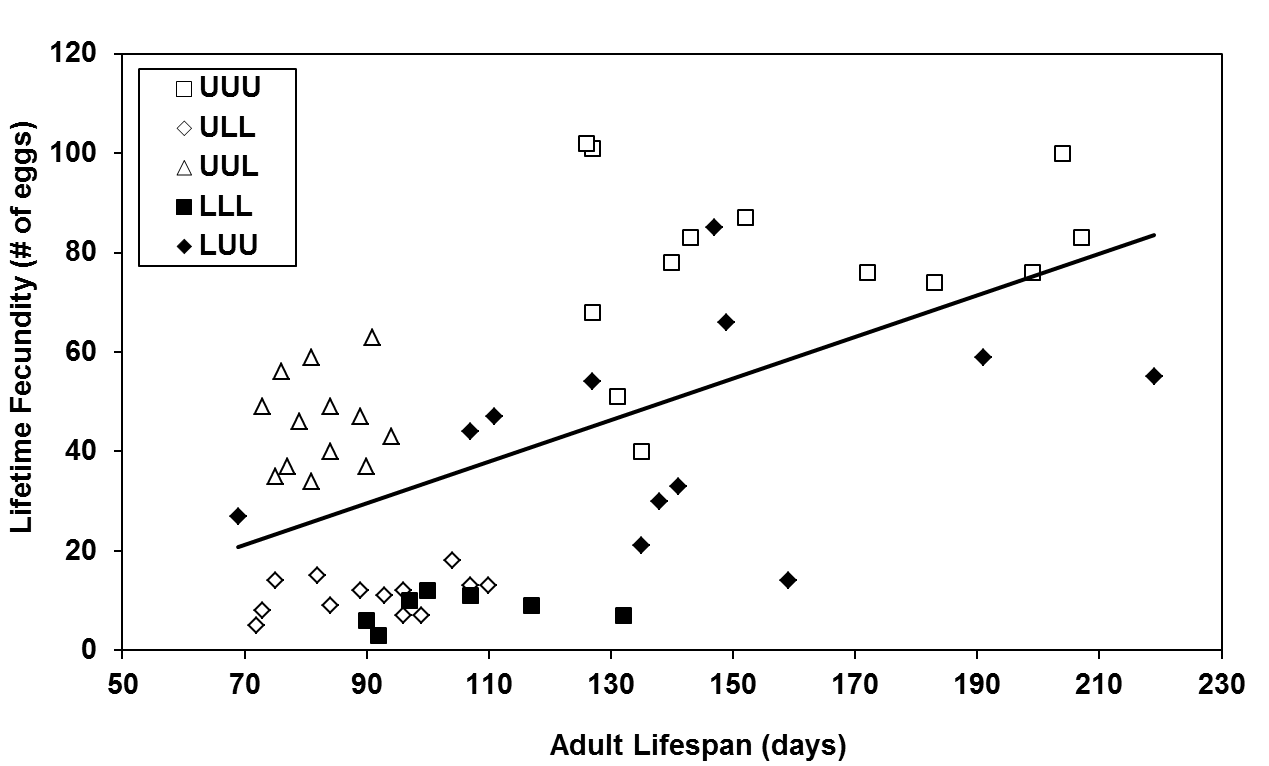


**y = 0.418x – 7.999**

***F_1,56_* = 25.67, *p* < 0.0001,**

**R^2^ = 0.302**

Figure S11. Relationship between fecundity and the duration of adult lifespan (days) for all insects that oviposited (*n* = 58) as determined by least squares linear regression. U = unlimited access to food, L = limited access to food.
