# Supplementary material for: Bridging Developmental Boundaries: Lifelong Dietary Patterns Modulate Life Histories in a Parthenogenetic Insect
Source: PLoS One. 2014 Nov 3;9(11):e111654. doi: 10.1371/journal.pone.0111654 (PMC4218793; doi:10.1371/journal.pone.0111654)
Supplement: Table S1 — The five dependent and 91 independent variables tested in stepwise linear regression analyses. (DOC) [file pone.0111654.s012.doc]

Table S1. The five dependent and 91 independent variables tested in stepwise linear regression analyses that yielded the models in Table 1.

| Dependent variables tested: |
| --- |
| Fecundity (A models) |
| Total number of eggs oviposited during first six days of reproductive lifespan (B models) |
| Adult lifespan (C models) |
| Reproductive lifespan (D models) |
| Total lifespan (E model) |

| Independent variables tested: |
| --- |
| Body mass at end of first instar (g) |
| Age at end of first instar (d) |
| Duration of first instar (d) |
| Specific growth rate in first instar (d^-1^) |
| Mass-specific intake in first instar (g/g/d) |
| Total intake in first instar (g) |
| Nitrogen assimilated in first instar (g) |
| Body mass at end of second instar (g) |
| Age at end of second instar (d) |
| Duration of second instar (d) |
| Specific growth rate in second instar (d^-1^) |
| Mass-specific intake in second instar (g/g/d) |
| Total intake in second instar (g) |
| Nitrogen assimilated in second instar (g) |
| Body mass at end of third instar (g) |
| Age at end of third instar (d) |
| Duration of third instar (d) |
| Specific growth rate in third instar (d^-1^) |
| Mass-specific intake in third instar (g/g/d) |
| Total intake in third instar (g) |
| Nitrogen assimilated in third instar (g) |
| Body mass at end of fourth instar (g) |
| Age at end of fourth instar (d) |
| Duration of fourth instar (d) |
| Specific growth rate in fourth instar (d^-1^) |
| Mass-specific intake in fourth instar (g/g/d) |
| Total intake in fourth instar (g) |
| Nitrogen assimilated in fourth instar (g) |
| Body mass at end of fifth instar (g) |
| Age at end of fifth instar (d) |
| Duration of fifth instar (d) |
| Specific growth rate in fifth instar (d^-1^) |
| Mass-specific intake in fifth instar (g/g/d)^δ^ |
| Total intake in fifth instar (g) |
| Nitrogen assimilated in fifth instar (g) |
| Body mass at end of sixth instar (g) |
| Age at end of sixth instar (d) |
| Duration of sixth instar (d) |
| Specific growth rate in sixth instar (d^-1^) |
| Mass-specific intake in sixth instar (g/g/d) |
| Total intake in sixth instar (g) |
| Nitrogen assimilated in sixth instar (g) |
| Actual:predicted body mass at the adult molt |
| Length at adult molt (mm) |
| Duration of pre-oviposition adult stage (d)^δ^ |
| Specific growth rate in pre-oviposition adult stage (d^-1^) |
| Mass-specific intake in pre-oviposition adult stage (g/g/d) |
| Total intake in pre-oviposition adult stage (g)^δ^ |
| Nitrogen assimilated in pre-oviposition adult stage (g)^δ^ |
| Body mass at first oviposition (g) |
| Length at first oviposition (mm) |
| Age at first oviposition (d) |
| Early egg output (total eggs oviposited during first six days of reproductive lifespan)^‡^ |
| Reproductive lifespan (d)^*‡δΨ ξ^ |
| Mass-specific intake during reproductive lifespan (g/g/d)^‡^ |
| Total intake during reproductive lifespan (g)^‡δΨ ξ^ |
| Nitrogen assimilated between first oviposition and death (g)^*‡δΨξ^ |
| Total intake in instars 1-4 (g) |
| Nitrogen assimilated in instars 1-4 (g) |
| Total intake in instars 1-6 (g) |
| Total intake in instars 2-6 (g) |
| Total intake in instars 3-6 (g) |
| Total intake in instars 4-6 (g) |
| Total intake in instars 5-6 (g) |
| Total nitrogen assimilated in instars 1-6 (g) |
| Total nitrogen assimilated in instars 2-6 (g) |
| Total nitrogen assimilated in instars 3-6 (g) |
| Total nitrogen assimilated in instars 4-6 (g) |
| Total nitrogen assimilated in instars 5-6 (g) |
| Total intake in instars 1-6 and pre-oviposition adult stage (g)^‡δ^ |
| Total intake in instars 2-6 and pre-oviposition adult stage (g)^‡δ^ |
| Total intake in instars 3-6 and pre-oviposition adult stage (g)^‡δ^ |
| Total intake in instars 4-6 and pre-oviposition adult stage (g)^‡δ^ |
| Total intake in instars 5-6 and pre-oviposition adult stage (g)^‡δ^ |
| Total intake in sixth instar and pre-oviposition adult stage (g)^δ^ |
| Total nitrogen assimilated in instars 1-6 and pre-oviposition adult stage (g)^δ^ |
| Total nitrogen assimilated in instars 2-6 and pre-oviposition adult stage (g)^δ^ |
| Total nitrogen assimilated in instars 3-6 and pre-oviposition adult stage (g)^δ^ |
| Total nitrogen assimilated in instars 4-6 and pre-oviposition adult stage (g)^δ^ |
| Total nitrogen assimilated in instars 5-6 and pre-oviposition adult stage (g)^δ^ |
| Total nitrogen assimilated in sixth instar and pre-oviposition adult stage (g)^δ^ |
| Total intake from hatch to end of reproductive lifespan (g)^‡δΨ^ |
| Total intake from beginning of fifth instar through reproductive lifespan (g) ^‡δΨ^ |
| Total intake in pre-oviposition adult stage and reproductive lifespan (g) ^‡δΨ^ |
| Time between beginning of fourth instar and adult molt (d) |
| Time between beginning of fifth instar and adult molt (d) |
| Metabolic rate in fourth instar (µL O_2_/hr/g)^θ^ |
| Metabolic rate in fifth instar (µL O_2_/hr/g)^θ^ |
| Metabolic rate in sixth instar (µL O_2_/hr/g)^θ^ |
| Metabolic rate in pre-oviposition adult stage (µL O_2_/hr/g)^θ^ |
| Metabolic rate in post-oviposition adult stage (µL O_2_/hr/g)^θ^ |

Note: Some variables were excluded from individual stepwise linear regression analyses due to reliance on the dependent variable being tested, irrelevance, and/or collinearity with a previously selected variable. Independent variables that were excluded from analysis of each dependent variable are indicated by ^*^ (for fecundity), ^‡^ (for total number of eggs oviposited within the first six days of the reproductive lifespan), ^δ^ (for adult lifespan), ^Ψ^ (for reproductive lifespan), and ^ξ^ (for total lifespan). ^θ^ Data from [58].
