# Supplementary material for: Bridging Developmental Boundaries: Lifelong Dietary Patterns Modulate Life Histories in a Parthenogenetic Insect
Source: PLoS One. 2014 Nov 3;9(11):e111654. doi: 10.1371/journal.pone.0111654 (PMC4218793; doi:10.1371/journal.pone.0111654)
Supplement: Table S2 — Total leaf dry mass consumed during each life-history stage. (DOC) [file pone.0111654.s013.doc]

Table S2. Total leaf dry mass (g, means ± standard errors) consumed during each life-history stage.

|  | UUU | ULL | UUL | LLL | LUU |
| --- | --- | --- | --- | --- | --- |
|  |  |  |  |  |  |
| Instar 1 | 0.0082 ± 0.0002^aa^ | 0.0080 ± 0.0002^aa^ | 0.0079 ± 0.0002^a^ | 0.0088 ± 0.0003^aa^ | 0.0084 ± 0.0004^a^ |
| Instar 2 | 0.0197 ± 0.0005^aa^ | 0.0188 ± 0.0004^aa^ | 0.0196 ± 0.0004^a^ | 0.0226 ± 0.0004^bb^ | 0.0223 ± 0.0005^b^ |
| Instar 3 | 0.0411 ± 0.0009^aa^ | 0.0403 ± 0.0008^aa^ | 0.0410 ± 0.0007^a^ | 0.0358 ± 0.0010^bb^ | 0.0352 ± 0.0011^b^ |
| Instar 4 | 0.0795 ± 0.0016^aa^ | 0.0802 ± 0.0013^aa^ | 0.0772 ± 0.0012^a^ | 0.0636 ± 0.0009^bb^ | 0.0606 ± 0.0021^b^ |
| Instar 5 | 0.1558 ± 0.0025^aa^ | 0.1438 ± 0.0018^bb^ | 0.1550 ± 0.0016^a^ | 0.1289 ± 0.0031^cc^ | 0.1061 ± 0.0035^d^ |
| Instar 6 | 0.3473 ± 0.0054^ab^ | 0.3912 ± 0.0098^cc^ | 0.3514 ± 0.0053^b^ | 0.3162 ± 0.0078^ad^ | 0.2792 ± 0.0122^d^ |
| Pre-Ov Adult | 0.5757 ± 0.3280^aa^ | 0.6500 ± 0.0293^ab^ | 0.5747 ± 0.0135^a^ | 0.7312 ± 0.0614^bb^ | 0.7529 ± 0.0491^b^ |
| Post-Ov Adult | 2.6454 ± 0.1859^aa^ | 0.4289 ± 0.0478^bb^ | 0.9439 ± 0.0328^c^ | 0.2287 ± 0.0242^dd^ | 1.5177 ± 0.1963^c^ |

Notes: U = unlimited access to food, L = limited access to food. Sample sizes: UUU *n* = 13, ULL *n* = 13, UUL *n* =13, LLL *n* = 19 juveniles and 7 adults, LUU *n* = 12. Values with different superscripts are significantly different among treatment groups within a row.
