# Supplementary material for: Bridging Developmental Boundaries: Lifelong Dietary Patterns Modulate Life Histories in a Parthenogenetic Insect
Source: PLoS One. 2014 Nov 3;9(11):e111654. doi: 10.1371/journal.pone.0111654 (PMC4218793; doi:10.1371/journal.pone.0111654)
Supplement: Table S3 — Statistical results for body mass and age at each life-history transition. (DOC) [file pone.0111654.s014.doc]

Table S3. Statistical results for body mass and age at each life-history transition.

|  | Omnibus *F* and *χ*^2^ | *p*-Values for Pairwise Comparisons Between Groups | | | | | | | | | |
| --- | --- | --- | --- | --- | --- | --- | --- | --- | --- | --- | --- |
|  |  | UUU/ULL | UUU/UUL | UUU/LLL | UUU/LUU | ULL/UUL | ULL/LLL | ULL/LUU | UUL/LLL | UUL/LUU | LLL/LUU |
| Body Mass |  |  |  |  |  |  |  |  |  |  |  |
| Hatch | *F_4,65_* = 1.00, *p* = 0.4140 | 0.780 | 0.780 | 0.999 | 1.000 | 1.000 | 0.560 | 0.804 | 0.560 | 0.804 | 0.999 |
| End of Instar 1 | *F_4,65_* = 21.67, *p* < 0.0001 | 1.000 | 0.999 | < 0.0010 | < 0.0010 | 1.000 | < 0.0010 | < 0.0010 | < 0.0001 | < 0.0001 | 0.998 |
| End of Instar 2 | *F_4,65_* = 108.34, *p* < 0.0001 | 0.827 | 0.999 | < 0.0001 | < 0.0001 | 0.926 | < 0.0001 | < 0.0001 | < 0.0001 | < 0.0001 | 1.000 |
| End of Instar 3 | *F_4,65_* = 115.37, *p* < 0.0001 | 0.998 | 1.000 | < 0.0001 | < 0.0001 | 0.997 | < 0.0001 | < 0.0001 | < 0.0001 | < 0.0001 | 1.000 |
| End of Instar 4 | *F_4,65_* = 269.35, *p* < 0.0001 | 0.996 | 0.970 | < 0.0001 | < 0.0001 | 0.854 | < 0.0001 | < 0.0001 | < 0.0001 | < 0.0001 | 0.999 |
| End of Instar 5 | *F_4,65_* = 260.84, *p* < 0.0001 | < 0.0010 | 0.998 | < 0.0001 | < 0.0001 | < 0.0010 | < 0.0001 | < 0.0001 | < 0.0001 | < 0.0001 | 0.002 |
| End of Instar 6 | *χ^2^* = 62.92, *p* < 0.0001 | < 0.0001 | 0.801 | < 0.0001 | < 0.0001 | < 0.0001 | < 0.0001 | < 0.0001 | < 0.0001 | < 0.0001 | < 0.0001 |
| 1st Oviposition | *F_4,53_* = 59.01, *p* < 0.0001 | < 0.0001 | 0.993 | < 0.0001 | 0.010 | < 0.0001 | < 0.0010 | < 0.0001 | < 0.0001 | 0.031 | < 0.0001 |
| Death | *F_4,53_* = 33.26, *p* < 0.0001 | < 0.0001 | 0.366 | < 0.0001 | 0.003 | < 0.0001 | 0.001 | 0.019 | < 0.0001 | 0.243 | < 0.0001 |
|  |  |  |  |  |  |  |  |  |  |  |  |
| Age |  |  |  |  |  |  |  |  |  |  |  |
| End of Instar 1 | *χ^2^* = 51.66, *p* < 0.0001 | 0.880 | 0.840 | < 0.0010 | < 0.0010 | 0.960 | < 0.0001 | < 0.0001 | < 0.0001 | < 0.0001 | 0.795 |
| End of Instar 2 | *F_4,65_* = 686.21, *p* < 0.0001 | 1.000 | 1.000 | < 0.0001 | < 0.0001 | 1.000 | < 0.0001 | < 0.0001 | < 0.0001 | < 0.0001 | 0.975 |
| End of Instar 3 | *F_4,65_* = 626.17, *p* < 0.0001 | 0.980 | 1.000 | < 0.0001 | < 0.0001 | 0.991 | < 0.0001 | < 0.0001 | < 0.0001 | < 0.0001 | 0.989 |
| End of Instar 4 | *F_4,65_* = 518.30, *p* < 0.0001 | 1.000 | 0.994 | < 0.0001 | < 0.0001 | 0.995 | < 0.0001 | < 0.0001 | < 0.0001 | < 0.0001 | 0.997 |
| End of Instar 5 | *F_4,65_* = 591.77, *p* < 0.0001 | < 0.0001 | 0.942 | < 0.0001 | < 0.0001 | < 0.0001 | < 0.0001 | < 0.0001 | < 0.0001 | < 0.0001 | < 0.0010 |
| End of Instar 6 | *F_4,65_* = 888.90, *p* < 0.0001 | < 0.0001 | 0.952 | < 0.0001 | < 0.0001 | < 0.0001 | < 0.0001 | < 0.0001 | < 0.0001 | < 0.0001 | < 0.0001 |
| 1st Oviposition | *χ^2^* = 50.46, *p* < 0.0001 | < 0.0001 | 0.840 | < 0.0001 | < 0.0001 | < 0.0001 | < 0.0001 | < 0.0001 | < 0.0001 | < 0.0001 | < 0.0001 |
| Death | *F_4,53_* = 95.65, *p* < 0.0001 | 0.031 | < 0.0001 | < 0.0001 | < 0.0010 | < 0.0001 | < 0.0001 | < 0.0001 | < 0.0001 | < 0.0001 | 0.360 |

Notes: U = unlimited access to food, L = limited access to food. Sample sizes: UUU *n* = 13, ULL *n* = 13, UUL *n* =13, LLL *n* = 19 juveniles and 7 adults, LUU *n* = 12. When *F* values are reported, parametric tests were used. When *χ^2^* values are reported, nonparametric tests were used.
