# Supplementary material for: Bridging Developmental Boundaries: Lifelong Dietary Patterns Modulate Life Histories in a Parthenogenetic Insect
Source: PLoS One. 2014 Nov 3;9(11):e111654. doi: 10.1371/journal.pone.0111654 (PMC4218793; doi:10.1371/journal.pone.0111654)
Supplement: Table S4 — Body condition at the adult molt, mass-corrected fecundity, number of ovarioles, and unfulfilled reproductive potential. (DOC) [file pone.0111654.s015.doc]

Table S4. Body condition (actual:predicted body mass) at the adult molt, fecundity corrected for body mass at first oviposition, number of ovarioles, and unfulfilled reproductive potential (means ± standard errors).

|  | Actual:Predicted Body Mass | Adjusted Mean Fecundity (# of eggs) | Number of Ovarioles | Unfulfilled Rep. Potential (# of eggs) |
| --- | --- | --- | --- | --- |
|  |  |  |  |  |
| UUU | 1.087 ± 0.016^a^ | 68.061 ± 4.386^aa^ | 53.38 ± 0.488 | 3.00 ± 0.954^ab^ |
| ULL | 0.977 ± 0.016^b^ | 22.204 ± 4.524^bb^ | 53.00 ± 0.453 | 0.85 ± 0.222^aa^ |
| UUL | 1.093 ± 0.020^a^ | 36.649 ± 4.190^bc^ | 53.23 ± 0.556 | 1.85 ± 0.390^ab^ |
| LLL | 0.995 ± 0.014^b^ | 26.929 ± 6.815^bc^ | 50.86 ± 1.262 | 5.57 ± 0.997^bb^ |
| LUU | 1.148 ± 0.013^a^ | 42.719 ± 3.579^cc^ | 51.33 ± 0.449 | 4.50 ± 1.104^ab^ |

Notes: U = unlimited access to food, L = limited access to food. Sample sizes for actual:predicted body mass: UUU *n* = 13, ULL *n* = 13, UUL *n* =13, LLL *n* = 19, LUU *n* = 12. Sample sizes for adjusted mean fecundity, number of ovarioles, and unfulfilled reproductive potential: UUU *n* = 13, ULL *n* = 13, UUL *n* =13, LLL *n* = 7, LUU *n* = 12. Values with different superscripts within a column are significantly different among treatment groups. The differences in mean number of ovarioles for groups LUU and LLL as compared to group UUU were marginally significant (*p* < 0.10).
